# Generalized blood vessel models for magnetic nanoparticle-based oncology: geometric and microfluidic properties

**DOI:** 10.1038/s41598-026-37348-7

**Published:** 2026-01-27

**Authors:** Daniel Fleischhauer, Samuel Schlicht, Dietmar Drummer

**Affiliations:** 1 Institute of Polymer Technology, Friedrich-Alexander-Universität Erlangen-Nürnberg, Am Weichselgarten 10, 91058 Erlangen, Germany; 2 SyMoCADS – Research Training Group 2950, 91058 Erlangen, Germany

**Keywords:** 3D printed microfluidics, Magnetic drug targeting, Particle distribution, Microfluidic test bench, Nanoparticles, Cancer, Engineering, Mathematics and computing, Nanoscience and technology, Physics

## Abstract

Superparamagnetic iron oxide nanoparticles (SPIONs) represent an emerging class of nanoparticles that face increasing applications in medicine, in particular in nanoparticle-based oncology. Their superparamagnetic properties allow for the magnetic steering and the interlinked targeted and localized delivery of pharmaceuticals. The development of nanoparticle-based therapies requires a deep understanding of geometry-hydrodynamics-adhesion interactions, motivating the generation of blood vessel models. The present work addresses the geometry-dependent propagation of SPIONs under magnetic steering through generalized, transferable geometries. Such geometries were derived based on generalized, statistical considerations of branch-dependent vessel diameters, yielding a reproducible and transferable testing environment independent of individual angiographic data. Based on stereolithographic additive manufacturing, fluidic models with varied blood vessel diameters and branching orders were manufactured and tested under varying magnetic steering conditions, injected SPION concentration, and flow rate. Through optical in situ measurements and complementary *ex situ* scanning electron microscopy, a significant influence of flow-regime-dependent hydrodynamic effects on magnetic steerability could be identified. Experimental findings suggest that while reduced flow rates were associated with locally laminar flows that promoted sedimentation and enabled limited magnetic redistribution of SPION-containing colloidal solutions at a magnetic flux density of B = 0.35 T, increased flow rates and interlinked unsteady flows were shown to impair the magnetically controlled, local SPION deposition. Hence, flow conditions present in larger arteries and bifurcations during SPION injection can be shown to significantly influence the quantitative magnetic steering of SPIONs in vascular-inspired fluidic channel structures, displaying enhanced local particle residence times and redistribution of SPIONs under low-flow conditions.

**Supplementary Information:**

The online version contains supplementary material available at 10.1038/s41598-026-37348-7.

## Introduction

Superparamagnetic iron oxide nanoparticles (SPIONs) increasingly enable targeted therapeutic interventions in oncology. The targeted delivery of therapeutics has the potential to increase treatment success by the prolonged accumulation in the tumor tissue while the exposure of the rest of the body to cytotoxic drugs is significantly reduced^1^. The superparamagnetism represents a significant prerequisite to the magnetic steering and localized delivery of therapeutic agents, thereby forming the methodological basis for therapies with minimized systemic side effects^2^. However, the effective clinical translation of SPION-based therapies relies on a fundamental understanding of nanoparticle transport phenomena within vascular networks, in particular concerning interactions governed by varying vascular geometries, locally present flow conditions, and the nanoparticle-wall interaction^3^. The experimental modeling of nanoparticle propagation in vascular systems has been shown to be inherently challenging due to the geometric complexity of native vascular geometries. Local particle concentration is affected by a variety of factors, including variability in diameter, branch angles, segment lengths, and hierarchical organization^4^. These geometric characteristics influence local hemodynamics such as shear forces and flow velocities alongside disturbed flow states, which directly impact nanoparticle trajectories, margination tendencies, and wall adhesion probabilities^5^. Moreover, vessel branching patterns could be observed to alter the local particle distributions and the propagation of particles throughout the vessels, with bifurcations frequently serving as regions of increased nanoparticle adhesion due to flow separation and reduced velocities^6^. Consequently, vascular geometry plays a pivotal role in nanoparticle-based therapies, yet the inherent variability and complexity of real biological vessel systems limit reproducibility and comparability of experimental results. While magnetic drug targeting strategies increasingly rely on collective particle effects and carrier systems to enhance magnetic responsiveness, the physical transport limits that govern magnetically actuated nanoparticle motion in complex branched-flow networks remain insufficiently constrained experimentally^7–9^.

To address these challenges, the development of generalized vascular models becomes increasingly significant. Generalization, in this context, refers to systematic abstraction and simplification of real vascular structures, maintaining representative and physiologically relevant geometric parameters, such as diameter ratios, branching angles, and fractal-like branching laws derived from empirical studies^10^. Generalized vascular models typically rely on well-established theoretical frameworks, such as Murray’s law, which describes relationships between vessel diameters, flow distribution, and metabolic efficiency^11^. Empirical investigations have also confirmed consistent geometric relationships, including scaling laws linking vessel length and radius, further enabling generalized vessel geometries applicable across various tissues and pathological conditions^12^.

While traditional experimental models are often highly specific as these reflect particular biological structures, generalized vascular models embed the potential to provide robust and transferable test environments. Such generalized models allow for systematic investigations of nanoparticle interactions and their oncologic application under reproducible and controlled yet biologically representative conditions. Additionally, generalized models have been described to enable the comparison of experimental data and facilitate validation and refinement of theoretical and computational models of nanoparticle transport, adhesion, and therapeutic efficacy^13^. Concerning the generation of such models, additive manufacturing (AM) techniques offer a versatile toolbox for realizing particularly complex generalized vascular structures due to their geometric flexibility. Arising from their achievable spatial resolution and advantageous smooth surfaces, Digital Light Processing (DLP), among other AM methods, has emerged as a frequently employed rapid prototyping process for complex channel networks. Thus, DLP enables the reproduction of generalizable and physiologically representative vascular patterns derived from statistical analyses of angiographic or morphological data, capturing essential characteristics without overly restrictive specificity^10^. Furthermore, the understanding nanoparticle transport and adhesion in vascular structures benefits from concepts drawn from molecular communication. In this analytical framework, nanoparticles represent information carriers whose distribution and retention within vascular networks depend on flow-mediated transport phenomena and local geometrical constraints^14^. In this regard, the propagation of nanoparticles in viscous media may furthermore be considered and mathematically described as the propagation of a message through a communication channel.

In the present work, a generalized, additively manufactured vascular-inspired modelling approach is introduced to systematically investigate SPION propagation and adhesion behavior. The approach leverages statistical and theoretical vascular geometry generalizations to create reproducible experimental environments inspired by statistical features reported for tumor-associated vascular geometries. By enabling controlled variation of geometric parameters and flow conditions, this generalized model approach provides fundamental insights critical for advancing nanoparticle-based therapeutic strategies in oncology.

## State of the Art

### Generalized modelling of vascular structures

The development of vascular network morphology is mainly governed by the efficiency of the transport of nutrients and oxygen. In 1926, based on Hagen-Poiseuille’s law Murray suggested that in a cylindrical tube under laminar flow for maximum efficiency the flow needs to be related to the cube of the radius^11^. Empirical investigations have also shown that the mean flow of blood volume per unit time *q* in a cylindrical vessel of radius *r* is proportional to its inner radius:

1 $$\:q\:\propto\:{r}^{\alpha\:}$$

The number *α* is called the radius exponent which Murray identified as three. At a bifurcating vessel the need of the preservation of flow at the junction leads to the following relationship between the size of the mother vessel and the daughter vessels^15,16^:

2 $$\:{r}_{0}^{\alpha\:}={r}_{1}^{\alpha\:}+{r}_{2}^{\alpha\:}$$

Based on Murray’s model, empirical investigations of hierarchical branching in various vascular tissues have reported mean radius exponents in the range between 2.0 and 3.0. Different studies and modelling approaches showed an influence of vessel size, elasticity and morphology on the observed radius exponent. While values from 2.0 to 2.3 were observed in the big proximal arteries, peripheral arteries smaller than 0.1 mm typically showed a value of α = 3. For elastic-muscular arteries in the range between the proximal arteries and the peripheral systemic arteries, based on the assumption of blood vessels being rigid cylindrical tubes, an optimal radius exponent of 2.7 was reported^15,16^.This value has been linked to the minimization of vascular wall thickness. Furthermore, also a relationship between the length of a vessel segment and its radius has been found. This links the two parameters by a proportionality factor Ω and an exponent κ which leads to the following expression^17^:

3 $$\:l\:=\:{\Omega\:}{r}^{\kappa\:}$$

For the exponent κ, studies have found values between 0.76 and 1.21. The fractal analysis of the vascular tree yielded values of Ω in a range of 4.6 to 17.6 depending on the tissue type^16^. In order to achieve the most energy efficient transport, several studies describe the mammalian blood vessel system as a space filling self-similar branching system^18,19^. This assumption requires that besides the proportionality of the radius that follows from Murray’s law also the segment length is proportional between branches exhibiting a constant scaling factor across the generations.

### Particle transport and deposition in viscous flows

Fluid transport in tubes is governed by mass conservation. For a fluid of constant density ρ, the volumetric flow rate remains constant along the flow path. The volumetric flow rate depends on the flow velocity and the tube’s cross-sectional area. By the continuity equation at a fixed volumetric flow rate, cross-sectional area and flow velocity are inversely proportional; as vessel radius decreases, flow velocity increases.

The transport and propagation of particles are governed by multiple factors. Foremost is advection, which describes the convective carriage of injected particles by the background flow via inertial effects^20,21^. Higher flow velocities generally yield faster downstream transport, implying that increasing flow rates or decreasing cross-sectional areas shorten the downstream transit time. At a given injection rate, increased flow velocities lower the fraction of injected volume within the background flow, reducing local particle concentration. In hierarchical branching networks, Murray’s law indicates that the cumulative surface area of all channels within a branching generation increases with branching level, producing progressively slower flow as branching complexity and the number of branchings, respectively, rises. The deceleration follows a hyperbolic trend, implying a relatively increased deceleration at low branching levels while it shows a diminishing trend as the number of branchings increases^22,23^. In addition to hydrodynamic factors, particle transport is governed by dispersion. Under laminar flow with no-slip at the wall, velocity decreases toward the wall and a parabolic profile develops. Particles follow the streamlines of this profile; particles near the centre travel downstream faster than those near the wall, generating a non-uniform transverse particle distribution. These concentration gradients, induced by the parabolic profile, drive dispersion within the channel. Along the longitudinal axis, diffusion mechanisms also induce particle dispersion^20,24^. The impact of dispersion on local particle distributions scales with effective flow velocity and channel diameter; higher velocities and larger diameters increase dispersion^25^. In hierarchical branching systems, macroscopic dispersion depends on local flow velocities and segment lengths. Variations in travel distance and residence time across segments enhance post-junction dispersion through differential arrival times of the flows. The effect intensifies with higher branching and network complexity. The local concentration at a given point along the channel results from the superposition of advection and dispersion^20,26^. Advection sets the drift of the particle cloud, while dispersion determines the temporal shape of the concentration profile. Together, they produce a skewed concentration-versus-time curve with a prolonged tail^27^. Other contributors to nanoparticle dispersion include diffusion via Brownian motion, shear induced lift forces and convective particle transport induced by disturbed flow^28^.

Beyond hydrodynamics and diffusion, gravitational effects influence particle propagation. Particles denser than the surrounding medium segregate and settle to the bottom of the channel, creating transverse concentration gradients. High particle volume fractions and low flow velocities amplify this effect, producing elevated near-bottom concentrations. These high concentrations promote creeping flow at the wall and are further modulated by particle–particle interactions. Higher velocities induce lift, increasing the fraction of particles transported in suspension^29^. At a bifurcation under laminar flow, gravity biases the partitioning, yielding an asymmetric distribution of particles between the daughter channels in the direction of the gravitational field.

### Nanoparticle-based oncology and clinical models

The main goal of magnetic drug targeting is the direction of the medication laden particles into the target tissue and the accumulation to support the uptake of the medication in the target region. The effectiveness of the therapy is depending on the uptake of particles by the surrounding tissue and therefore strongly determined by the flow related particle transport, particle deposition due to fluidic factors and particle-wall-interactions as well as the effectiveness of the magnetic field for guiding to and holding the particles inside the tumor^1^. Magnetic drug targeting was previously performed on tumors in rabbits and mice. Those in vivo studies showed promising results in the effective of therapy of tumors using magnetic nanoparticles^30–33^. However, those animal studies are not suitable of the study of hydrodynamic particle transport phenomena. In order to understand this complex interplay of factors various experimental models have been applied to investigate the determinants of successful magnetic steering strategies. In vitro tests have investigated the influence of applied magnetic fields on cellular particle uptake^33^. These tests were performed with cell cultures in a particle bearing medium. Application of a magnetic field has shown the potential of magnetic fields to enhance the delivery of particles into the cell and accumulation in a target region^34^. These studies however neglect the influence of flow and geometry on the particle response to magnetic fields. Investigations on the influence of flow conditions and magnet position and strength on the magnetic accumulation were conducted using ex vivo tubular segments of umbilical arteries or bovine carotid arteries^35,36^. Based on histologic measurements, those investigations showed the accumulation of particles in the arterial wall depending on the strength of the magnetic field. Steering experiments were also performed with synthetic geometric models of straight or bifurcating arteries under physiological flow conditions. The models often consist of glass or silicone tubes and exhibit a Y-branch. In these experiments, the magnetic field and position were related to the steering efficiency and trapping capability of the steering system^38–40^. However, the experimental models found in literature are confined to a singular branching the size of a bigger artery, neglecting the high complexity and the heterogeneous flow conditions of multiple branched networks as well as the effect of decreasing vessel diameter on the particle behaviour^41^. Furthermore, tumor specific features such as occlusions, strongly bent vessels or a high number of wall defects that lead to flow disturbances and heterogeneous blood perfusion is not duly represented in current models^41,42^. To further increase the effectiveness of newly developed particle steering methods, there is a need for an understanding of the influence of the vascular structure on the particle propagation through more complex structures and the resulting particle distribution within the network. These represent important determinants for the effective accumulation and trapping of particles within the tumor, requiring the development of novel generalized, reproducible experimental flow channel models that reflect the branching network complexity.

## Materials and methods

### Model design

Based on the self-similar fractal geometric patterns in vascular systems, a statistically derived physiologic vascular geometry was derived in order to provide a realistic generalized modelling of fluidic processes. At the same time clinical data for sizes of tumor blood vessels in the tumor feeding system and in the vascular bed found in the literature were taken as boundary conditions. MRI studies of mammary carcinoma show a diameter of the tumor feeding vessels in the range of a few millimetres^43^. Based on these studies for the channel entering the model, a diameter of 2.5 mm was chosen. Less et al. measured the sizes of blood vessels in the microvascular environment of breast tumors and correlated the blood vessel diameter to the branching order. The study showed a reduction of blood vessel size from arteries in the range of 100 μm down to the capillary bed that exhibited a similar development as would be expected on the basis of Murray’s law. These experimentally derived values acted as a starting point for the determination of the channel diameters in hierarchically branched channel structures. A moving average with a window size of 3 was applied to empirical data reported by Less et al.^44^ in order to reduce the impact of statistical noise on the data and the derived generalization. The diameters of larger arteries of the macroscopic vessel system were extrapolated using Murray’s law with an exponent of α = 2.7 until the reference diameter diameter of 2.5 millimeters for the tumor feeding vessel was reached. For the sake of simplicity and generalizability, a symmetric branching with an angle of 120 degrees was applied. With the symmetrical branching Murray’s law was simplified to r_0_^2.7^ = 2r_1_^2.7^. The segment length corresponding to the branching order was calculated by using the empirical relation that the length of the segment in a hierarchically branched blood vessel system is proportional to the blood vessel radius^16,18^. The length of the initial segment was also chosen on basis of studies of the tumor feeding system where an average length of the main feeding vessel of 12.3 mm was found^43^. Under the assumption of self-similarity and the previously described proportionality of the blood vessel segment length to the corresponding radius, the segment length can be calculated as a function of the previous segment length and the proportionality of the blood vessel radii. By using these relationships, the following relationship was applied to the proportionality of the segment lengths. As the proportionality cancels out the factor Ω, the following relationship was derived for the segment length of the k^th^ branching order:

4 $$\:{l}_{k}=\:{l}_{k+1}\:({\frac{{r}_{k}}{{r}_{k+1}})}^{\kappa\:}$$

**Fig. 1:**
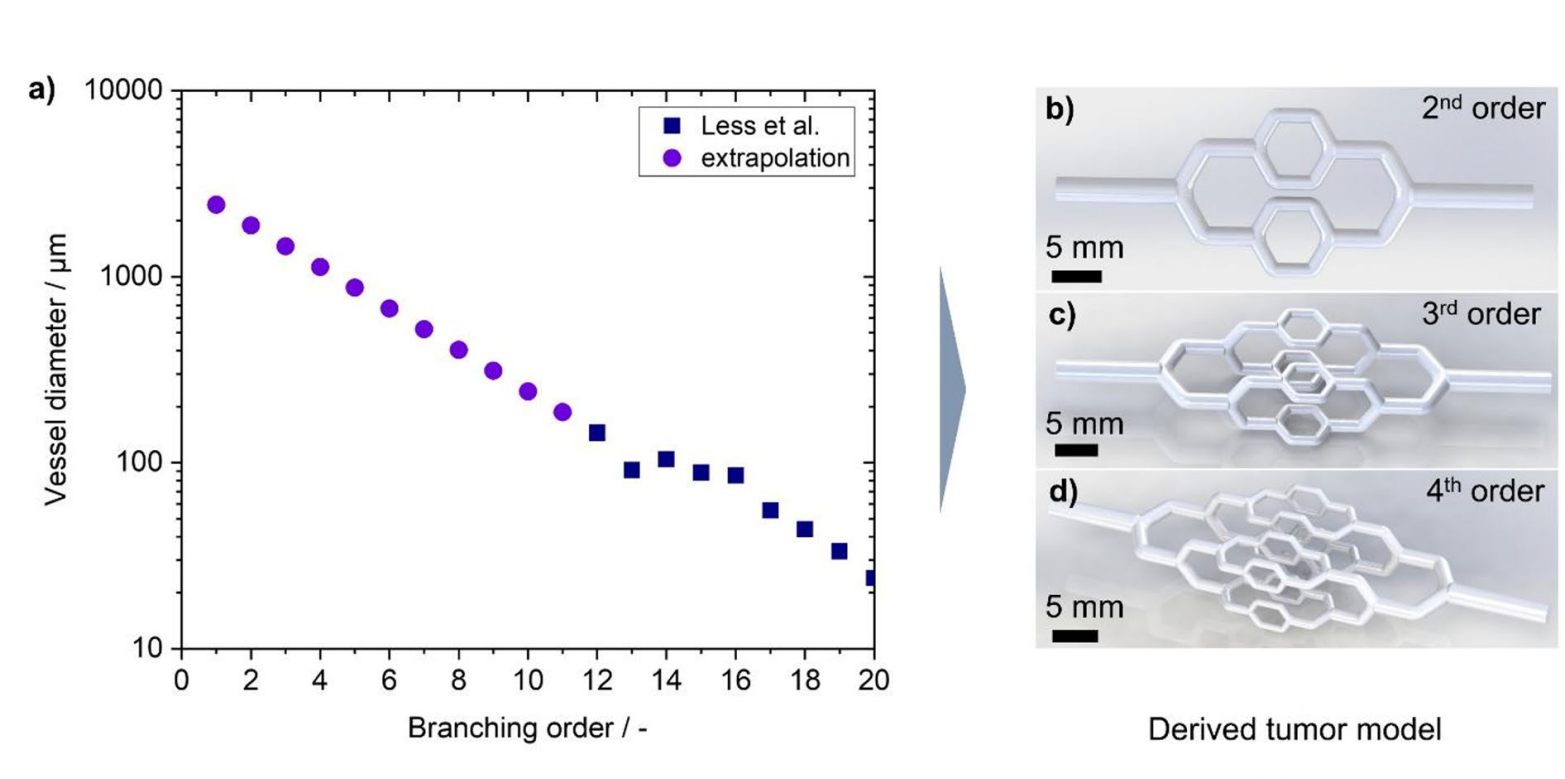
Derivation of cross-scale tumor-inspired geometries based on experimental data gathered by Less et al.^44^ and Murray’s law. **a)** The extrapolation of clinical data from Less et al.^44^ to higher vessel diameters according to Murray’s law. **b-d)** derived tumor-inspired model geometries for 2nd, 3rd and 4th branching order.

**Fig. 2:**
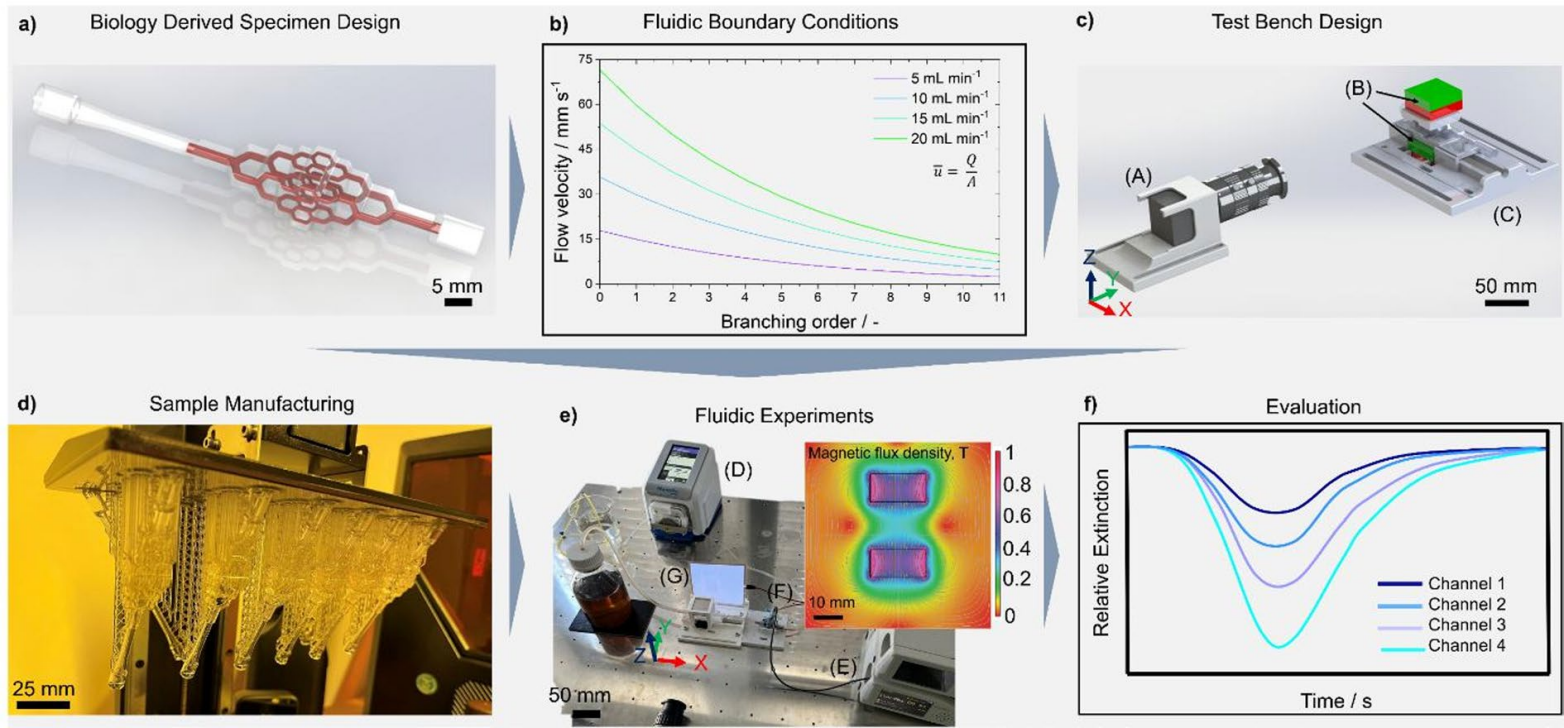
Overview of development and implementation of experimental models and fluidic testing. **(a)** Exemplary CAD-rendering of the 4th -order specimen for fluidic testing. The vascular-inspired channel geometry is highlighted in red. **(b)** Change of the average flow velocity with increasing branching order for different applied flow rates calculated from the continuity equation. **(c)** Design of the test bench and measurement setup. Visualizing the position of the specimens relative to the camera setup (A) and the magnets (B) on a fixture (C). **d)** Samples attached to the 3D printer’s platform after manufacturing. **e)** Overview of the test bench setup in the lab. Consisting of a peristaltic pump (D) providing the background flow and a syringe pump (E) providing the particle injection via a T-piece (F). The transmitting light was provided by a photo-luminecent backlight (G). On the side an exemplary plot of the magnetic flux density is provided, simulated using COMSOL CAE Software. **f)** Schematic extinction curves over time caused by particles passing through the channels retrieved from the measurements.

As boundary condition for the segment length, l_0_ = 12 mm was chosen according to the data obtained for the feeding blood vessel of mammary carcinoma^43^. For the exponent κ, a value of 1 was specified, based on the range of values found in statistical evaluation of clinical specimens by^17^. With the assumption of symmetrical branching, the segment length of both vessel branches was specified as equal. Based on the fractal nature of the branching tree, single modular branching elements were designed that can be connected to create samples with arbitrary branching complexity. To connect the vascular models to the periphery, a connector was added where the silicone tube could be simply plugged in to provide a seamless connection of the specimen and the test bench. Right after, the channel diameter was steadily reduced to the size of the model entrance. Based on these relationships, models with different branching complexity were developed. Models with two, three and four branching orders were designed. In the double-branched models, the branches were all on the same plane. In the triple- and quadruple-branched models, the segments were arranged such that the branching plane of one generation of branches was rotated 90 degrees to the previous one. Thus, increased branching levels also exhibited a controlled expansion in three dimensions. The positives of the branching structures derived by this approach are depicted on the right side of Fig. 1. To create a manufacturable channel structure, the negatives of the channels were then incorporated into a solid body to form channels incorporated into a matrix of surrounding material. An exemplary CAD-rendering of the 4th-order model is visualized in Fig. 2a) with the flow channel geometry highlighted in red. The resulting models were subsequently manufactured on a 3D-printer from UV curable acrylate resin. To ensure the visibility of the channels, a distance of 1 mm between the channel and the outer surface was established independent of the branching order. For each branching level, channels of the highest respective branching order could be observed under constant transmissive conditions.

### Sample manufacturing

Specimens with two, three and four branching generations were additively manufactured by Digital Light Processing (DLP). The specimens were printed using a transparent, commercially available standard resin (Anycubic High Clear Resin, Anycubic Technology Co., Ltd., Shenzhen, China) on a DLP 3D printer of type Phrozen Sonic Mighty 12k (Phrozen Tech Co., Ltd., Hsinchu City, Taiwan) with a pixel size of 19 × 24 µm^2^. Specimens were placed such that the channels were oriented at an angle of 60 degrees with respect to the building platform. The layer height was set to 25 μm and the exposure time was set to 2.7 s. After printing, obtained specimens were thoroughly cleaned with isopropanol. Residual resin within the channel was purged by flushing the channel structure with isopropanol multiple times. After this cleaning step, the specimens were submerged in isopropanol and ultrasonically cleaned for 15 min. After the cleaning the specimens were dried and remaining isopropanol was removed from the channels using compressed air. The dried vascular models then underwent UV post curing for 10 min at room temperature.

### Fluidic testing

For the experimental investigation of SPION propagation and adhesion in the vascular models, a test bench was set up. The testing setup was fed by a peristaltic pump placed upstream of the channel providing the background flow that simulates the blood flow. In the present work flow rates of 5, 10, 15 and 20 mL min^− 1^ were applied by this pump. At these flow rates the pump, the pump delivered speeds of 32.97, 65.93, 98.9 and 131.22 revolutions per minute, respectively. The impeller of the peristaltic pump is equipped with six rollers that deform a hose and thus deliver the fluid feed. This results in a stroke of 197.82, 395.58, 593.4 and 791.22 strokes per minute, respectively. The samples were made of a rigid material, which meant that pulsation was also present here. For a proof of concept, distilled water was chosen as a background medium. The liquid containing the SPIONs was injected by a syringe pump 50 mm before the vascular model entrance through a T-piece consisting of multiple three-way valves. The model was connected to the periphery of the test bench with a 50 mm long silicone tube with an inner diameter of 4 mm, plugged into the connector of the specimen. At the exit of the specimen, a tube with the same diameter was plugged in, that led into the waste reservoir. The model was placed on specially designed holders to enable a reproducible position within the setup with respect to the magnets and the measurement system. The length of the holder was chosen to align the sample with the magnet and camera when it is pushed until the edge of the guidance. The sample was longitudinally aligned with the x-axis, resulting in the flow at the entrance pointing to the negative x-direction. The channels that were evaluated were aligned with the x-z-plane. To prevent movement during the measurement, the baseplate and holder were designed in such a way that the components locked by friction. The plate also provided fixtures and guiding for two cuboid NdFeB magnets with a dimension of 30 × 30 × 15 mm^3^. The magnets were placed above and below the specimen with the direction of magnetization in z-direction. The fixture ensured a distance of the magnets of 25 mm, such that at the centre of the specimen, a magnetic flux density of a few hundred mT was present. The experimental setup is visualized in Fig. 2c) and e). The sample was placed such that the centre of the magnets aligned with the geometric centre of the specimens. Towards the edges, the flux density reduced, leading to a hourglass-shaped magnetic field, schematically shown in Fig. 2. The resulting field gradient forced the particles inside the specimen and towards the magnet. At a distance of 150 mm from the sample surface, a camera of the type Basler BA2-a2A5320-23ucB was placed to record the particles passing through the channels, applying a frame rate of 25 Hz, using a lens with a focal length of 50 mm. An electroluminescent diffuse background lighting was installed on the opposite side of the sample to provide a reproducible diffuse optical reference.

### System properties

The nanoparticles for the injection were synthesized and provided by the Section of Experimental Oncology and Nanomedicine (SEON) of the university hospital Erlangen, Germany. The particles were produced by precipitation in an aqueous solution of Fe(II) and Fe(III) salts under stirring through the slow addition of aqueous ammonia solution. For colloidal stabilization, the particles were then coated with lauric acid through the addition of lauric acid dissolved in acetone. Following the purification by means of dialysis, the particles were dispersed in water with a particle concentration of 15.67 mg mL^− 1^. The average hydrodynamic diameter of the particles was 40.45 nm with a polydispersity index of 0.218, obtained through dynamic light scattering. The particles showed a zeta-potential of −44.32 mV and a magnetic susceptibility of 0.0061794, that was used for calculation of magnetophoretic force on single particles, non-interacting particles. Supplementary Fig. S1 displays scanning electron micrographs of the clustering of particles following the drying of colloidal solutions on an object slide. From the initial particle concentration, the fluid was further diluted in distilled water to the targeted concentrations for the testing. Double distilled water was used for the background flow.

### Experimental procedure

The particle propagation was observed in terms of variation of the model branching complexity, flow rate of the background flow, the particle concentration in the injected fluid and the magnetic field. The complexity of the model was increased from two to three and four branching generations. The background flow provided by the peristaltic pump was provided with 5, 10, 15, and 20 mL min^− 1^. by the syringe pump, a fixed volume of 0.1 mL SPIONs was injected at a fixed flow rate of 10 mL min^− 1^. The particle concentration in the injected fluid was 5, 7.5, and 10 mg mL^− 1^. In order to investigate the effect of these boundary conditions on the effectiveness of magnetic steering, for each parameter the testing was performed without the application of a magnetic field and under magnetic steering conditions. Before every experiment, the feeding tube and the injecting tube were filled until the T-connector. Prior to fluidic testing, a calibration of the pump was performed to ensure constant fluidic conditions. After setup and calibration, the specimen was connected to the periphery via a 50 mm silicone tube segment with an inner diameter of 4 mm. Likewise, the exiting tube with the same diameter was fixed to the specimen. The specimen was then filled by the peristaltic pump. To support the purging of air bubbles, the specimen was then flushed thoroughly until no bubbles were present in the tubing and specimen. For the investigation of localized particle accumulations, five SPION injections were performed, accumulating to a total colloidal volume of 0.5 mL. Prior to each experiment, the background flow was established for 30 s prior to the first injection. Following the initial injection, further injections were performed in a temporal distance of 120 s. Following the last injection, the background flow was maintained for 40 s. Following fluidic testing, the tubing was drained of the liquid with the help of the peristaltic pump by removing the hose from the reservoir. In the cases of magnetic steering during testing, purging and the removal of liquid fractions was conducted under magnetic influence. The sample was subsequently removed from the test bench and the channels were purged of residual fluid using compressed air.

### Imaging analysis

The measurements conducted with the camera were saved as a sequence of images in the uncompressed Tiff-format. Using a Python script, recorded imaging sequences were subjected to low pass filtering using a Gaussian and converted to an 8-bit grayscale image. For the extraction of the local extinction profiles, regions of interest (ROIs) were defined. The ROIs had a size of 10 × 10 px^2^ and were further divided into four equally sized squares of 5 × 5 px^2^. The ROIs were defined in the centres of the straight segments at the highest branching level. For every frame of a measurement, for each of those sub regions, the mean and standard deviation of the pixel value was calculated. Additionally, the mean and standard deviation of the values of the four sub-regions was calculated. The pixel values of all ROIs were then normalized, yielding the relative pixel extinction in every ROI. As the grayscale value of a pixel is related to the presence of particles in that location^45,46^, derived extinction curves are applied for qualitative evaluation of the particle distribution across different channels and the retention time of the particles within a channel. As no calibration for the relation of particle concentration to the grayscale values was applied, it should be noted that the resulting curves are purely qualitative. The applied methodology is schematically summarized in Fig. 2.

### Confocal microscopy

In order to investigate the quality of the surfaces and feature depiction, the channels were cut open following microfluidic testing in the respective plane of the branching. The open channels were then investigated under the laser scanning microscope. Images were taken of the straight segments, curved segments and the branching, to evaluate the presence of defects and the influence of the manufacturing process on the printing quality. At the bottom of the channel, further confocal measurements of the surface roughness were performed. For this purpose, the surface profile was measured along ten lines along the longitudinal axis of the channel and the height profile was measured. Based on this surface profile, the average surface roughness R_a_ was derived as the mean value.

### Scanning electron microscopy

Samples undergoing the experimental procedure of five particle injection cycles were cut open in the plane of highest branching. Then, a thin platinum layer was sputtered onto the surface in order to increase the conductivity of the surface. The scanning electron micrographs were performed with a scanning electron microscope of Type Zeiss Gemini (Zeiss Microscopy GmbH, Oberkochen, Germany) using an acceleration voltage of 3 kV. Particle clusters at the bottom of the channels were identified and imaged using the InLens detector.

## Results

### Geometric characteristics of generalized models

In this study, based on the aforementioned theoretical and empirical relations of diameter after branching, segment length and branching angle, models inspired by vascular structures exhibiting different branching complexities were derived. The theoretical basis for the development of the structure was given by Murray’s law with a radius exponent of 2.7, which was empirically found for peripheral blood vessel systems. Breast cancer data by Less et al.^44^ for the micro vascular system in mammary carcinoma, smoothed by a moving average with a window size of 3 branching orders, was used as a starting point. The data was then extrapolated with Murray’s law, yielding a propagation of blood vessel diameter shown in Fig. 1 from the tumor entrance down to the capillary bed. As a simplifying assumption, the symmetry of the branching was assumed, leading to daughter vessels of equal size and simplifying Murray’s equation to$\:\:{r}_{0}^{2.7}=\:{r}_{1}^{2.7}+\:{r}_{2}^{2.7}$. As symmetrical branching was assumed, the branching angle was set to 120 degrees. Figure 1 displays a satisfactory transition between empirical tumor data and the applied extrapolation, indicating that Murray’s law represents a reasonable assumption for the propagation of blood vessel size. Angiography studies provided the upper boundary condition for the derived model geometry at about 2.5 mm based on angiography measurements of tumor feeding systems of mammary carcinoma^43^. Three different models were designed with progressing complexity exhibiting two, three and four branching generations, reflecting the vascular macrostructure of the first few branching generations after the tumor entrance. The simplest model exhibited a second order branching in one plane, which was used as a baseline. More complex models of the 3rd and 4th order are exhibiting a three-dimensional layout with the plane of the segment in each generation turned 90° to the segment before. The models consistently showed open channels after manufacturing without signs of residual, adhering resin. The outer surfaces that were not supported displayed homogeneous topographies without a macroscopically visible staircase effect, caused by the layer-by-layer manufacturing. The inner surface of the channels displays continuous features on a macroscopic level. Independent of the applied branching order, a characteristic staircase pattern is observed under scanning electron microscopy, likewise induced by the layer-wise manufacturing. Depending on the orientation of the channel during building, the resulting surface patterns show a different direction within the channel. Due to the upright orientation and the 60° angle, the patterns show a transverse orientation in the straight segments in proximity to the bifurcation. Supplementary Fig. S2 displays the surface roughness R_a_ correlated with the channel size. The measured channels correspond to the straight segments after the bifurcation. The surface roughness measured throughout the models was in the range between R_a_ = 5.7 and R_a_ = 7.7 μm and on the same level for all channel diameters. This shows that the DLP process provides a consistent surface quality for all channels independent of their diameter and the branching complexity. Moreover, these findings indicate that the channels are sufficiently cleaned from resin that was trapped in the channels after building during post processing. Considering the proximity of bifurcations, no significant changes in the surface roughness can be observed. However, the specimens exhibit a steep slope at the transition from the straight segment to the branching. This site could be prone to enhanced particle deposition due to the development of local eddies. The height difference of this slope is more pronounced for larger channels. The ratio of the slope height and the tube is nearly the same for all branches as the ratio between channel diameters is the same. The curvatures show a good depiction accuracy with no visible artefacts related to the discrete pixel exposure by the LCD-projector. This corroborates the suitability of the DLP process applied to provide an accurate feature depiction and high surface quality for all investigated vascular-inspired geometries independent of channel size. Depending on the proximity to bifurcations and corresponding topological variations, manufacturing-induced grooves show a different orientation with respect to the channel axis.

### Macroscopic transient nanoparticle distribution

**Fig. 3:**
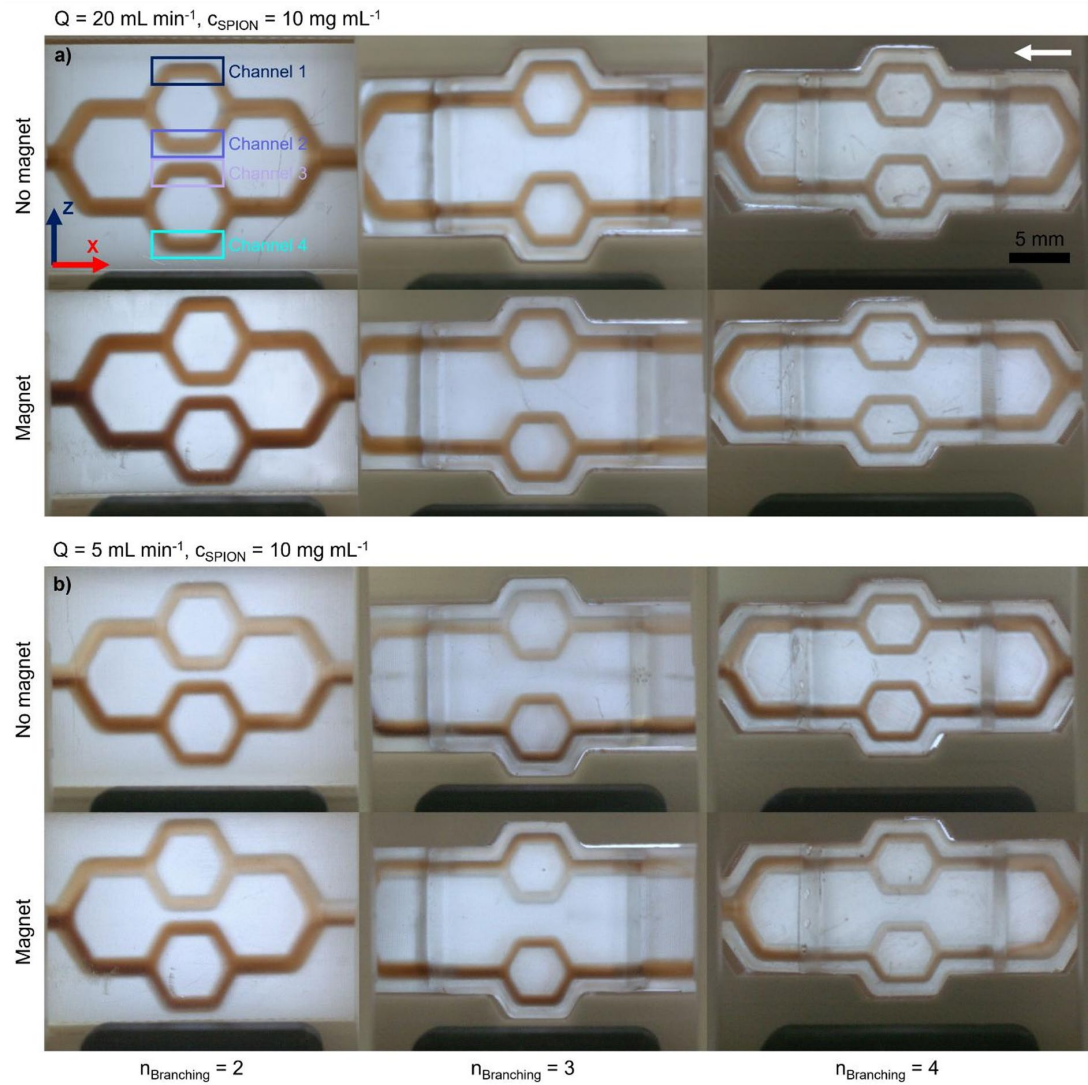
Particle distribution in the channel structures depending on the branching complexity with and without the influence of a magnetic field; exemplary denomination of the channel nomenclature. The white arrow indicates the direction of flow. **(a)** At a background flow rate of 20 mL min^−1^. **(b)** At a background flow rate of 5 mL min^−1^. Magnets were placed above and below the specimens on the z-axis with the direction of magnetization of both magnets corresponding to the direction of the z-axis.

Nanoparticle propagation through the channels was assessed visually across varying flow conditions and in the presence or absence of a magnetic field. In addition, localized, time-dependent particle presence was qualitatively assessed by relative pixel extinction. Figure 3 displays the particle distributions in the channel structures at the time of the extinction maximum in the highest-order segments for background flows of 20 mL min^− 1^ and 5 mL min^− 1^, respectively. The comparison of these flow regimes demonstrates that particle distribution within the channel network is strongly governed by the hydrodynamic conditions. In the 2nd-order structure, high flow rates result in a largely homogeneous propagation of particles throughout the network, both with and without magnetic actuation. In contrast, reducing the flow rate leads to pronounced particle sedimentation in the inlet channel. The resulting stratified particle population is subsequently leading to a preferential particle presence in the lower channels.

At a flow rate of 5 mL min^− 1^, at the entrance of the 2nd-order samples, the lower part of the channel appears darker than the upper part, indicating sedimentation of the particles toward the channel bottom. The sedimentation is already observable immediately after injection. As shown in S3, at 5 mL min^− 1^, particle volumes show sedimentation along the flow path and sink toward the bottom of the channel. However, after the branching the particles seem to be more homogeneously distributed within the channel. With increasing flow rate, the flow profile becomes more perturbed, and the development of injection-induced perturbance is observable even at 10 mL min^− 1^. At 20 mL min^− 1^, a uniform, well-mixed particle cloud is visible after injection. Further downstream, particle sedimentation also begins slowly to occur. At a flow rate of 5 mL min^− 1^, in the 3rd- and 4th-order models, the application of the magnet produces visible differences in particle distribution. Without the magnetic field, the uppermost channel of the 3rd-order model is least perfused with particles; with the magnet applied, more particles are present in this channel. In the 4th-order model, the two inner channels show visibly fewer particles. More particles seem to be present in the outer channels toward the magnets. A radial gradient toward the magnet is also observed. The apparent effect under magnetic conditions is greatest in the 3rd- and 4th-order models, where slower flow promotes geometry-dependent settling even in conditions without a magnet. However, images also show that in all cases a very low amount of particles is subject to magnetic effects.

**Fig. 4:**
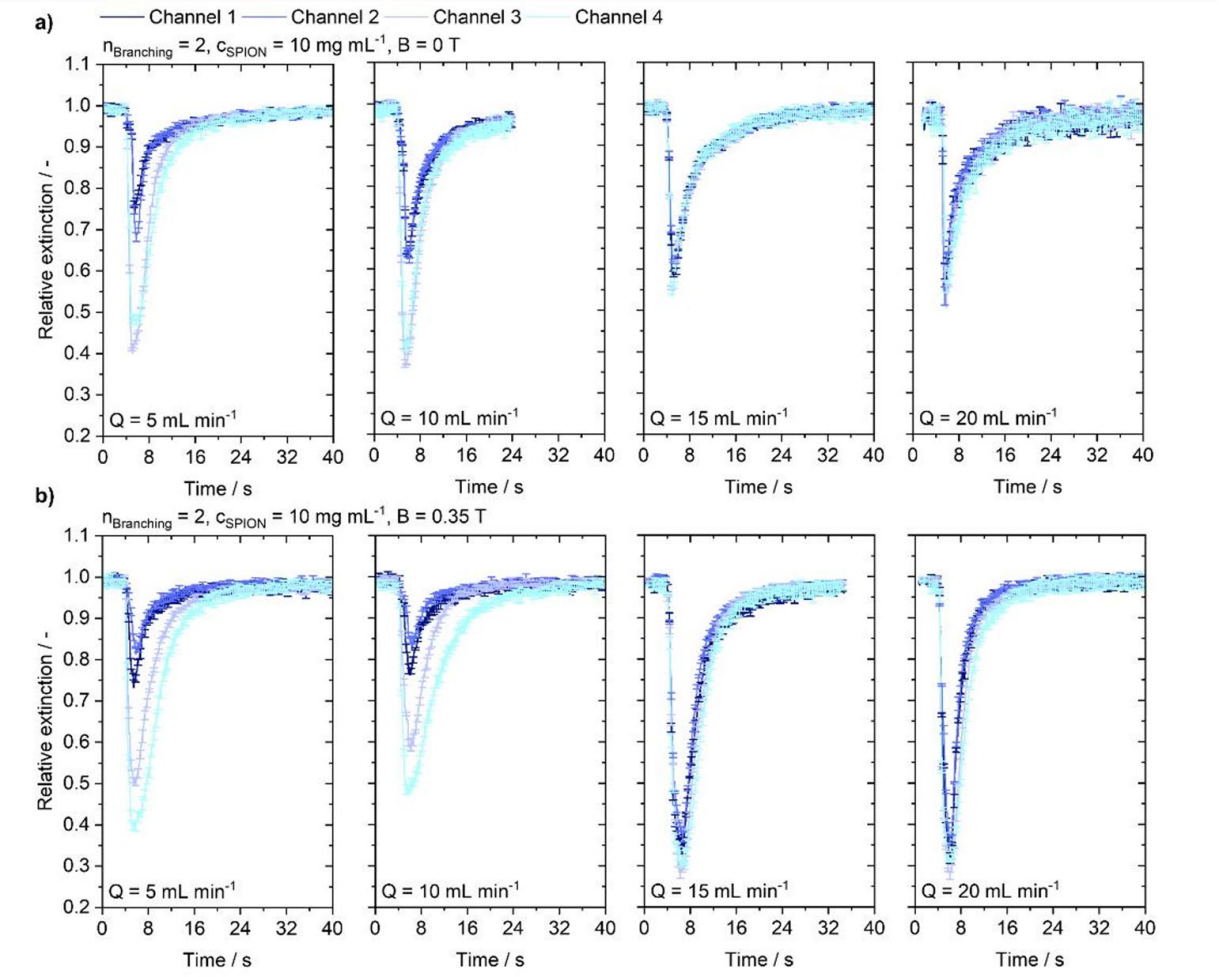
Flow-dependent extinction profile at 2nd-order branching injecting a particle suspension with a concentration of 10 mg mL^−1^ into the channel under varying flow conditions of 5, 10, 15 and 20 mL min^−1^. **(a)** Without the influence of a magnetic field. **(b)** Under magnetic steering conditions.

**Fig. 5:**
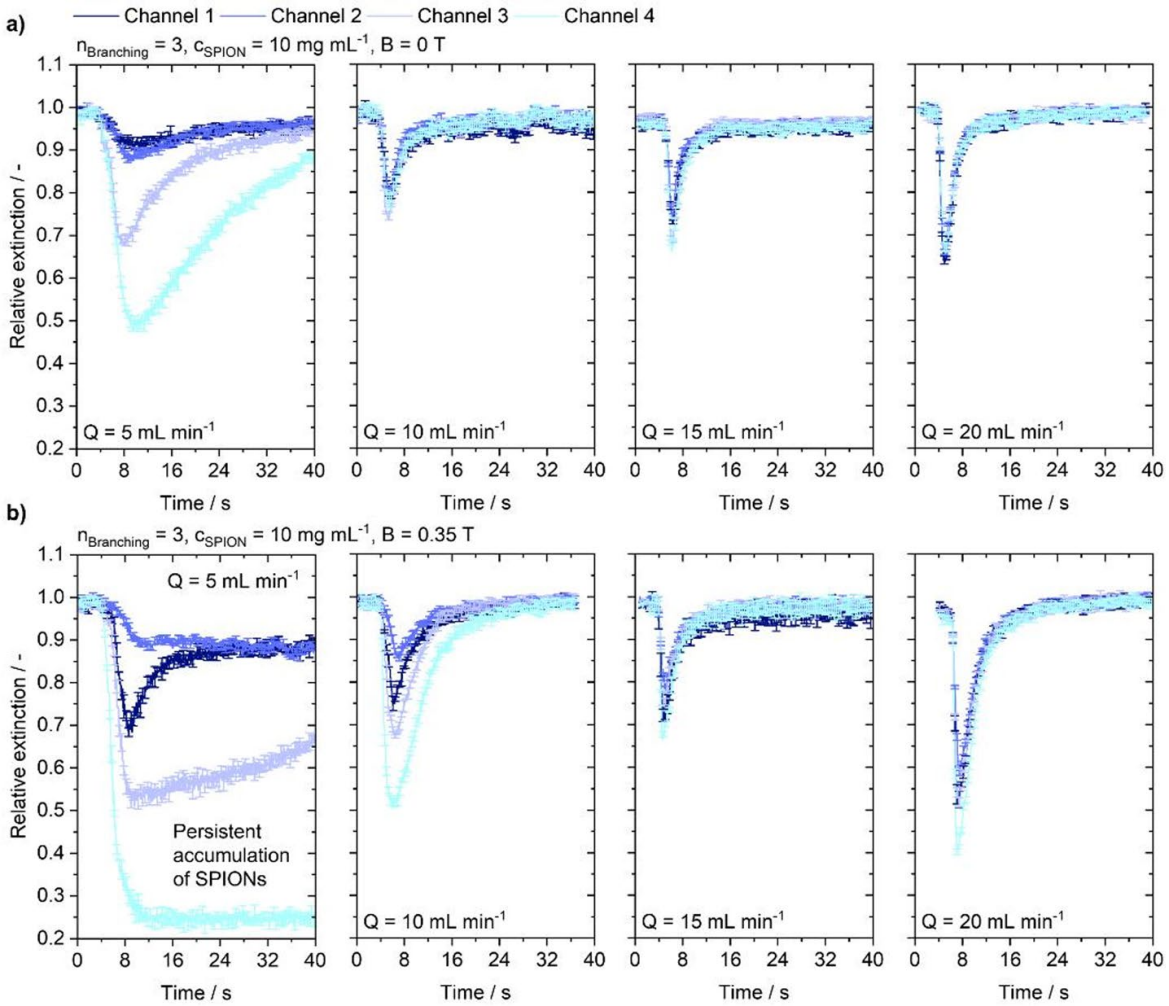
Flow-dependent extinction profile at 3rd-order branching injecting a particle suspension with a concentration of 10 mg mL^−1^ into the channel under varying flow conditions of 5, 10, 15 and 20 mL min^−1^. **(a)** Without the influence of a magnetic field. **(b)** Under magnetic steering conditions.

**Fig. 6:**
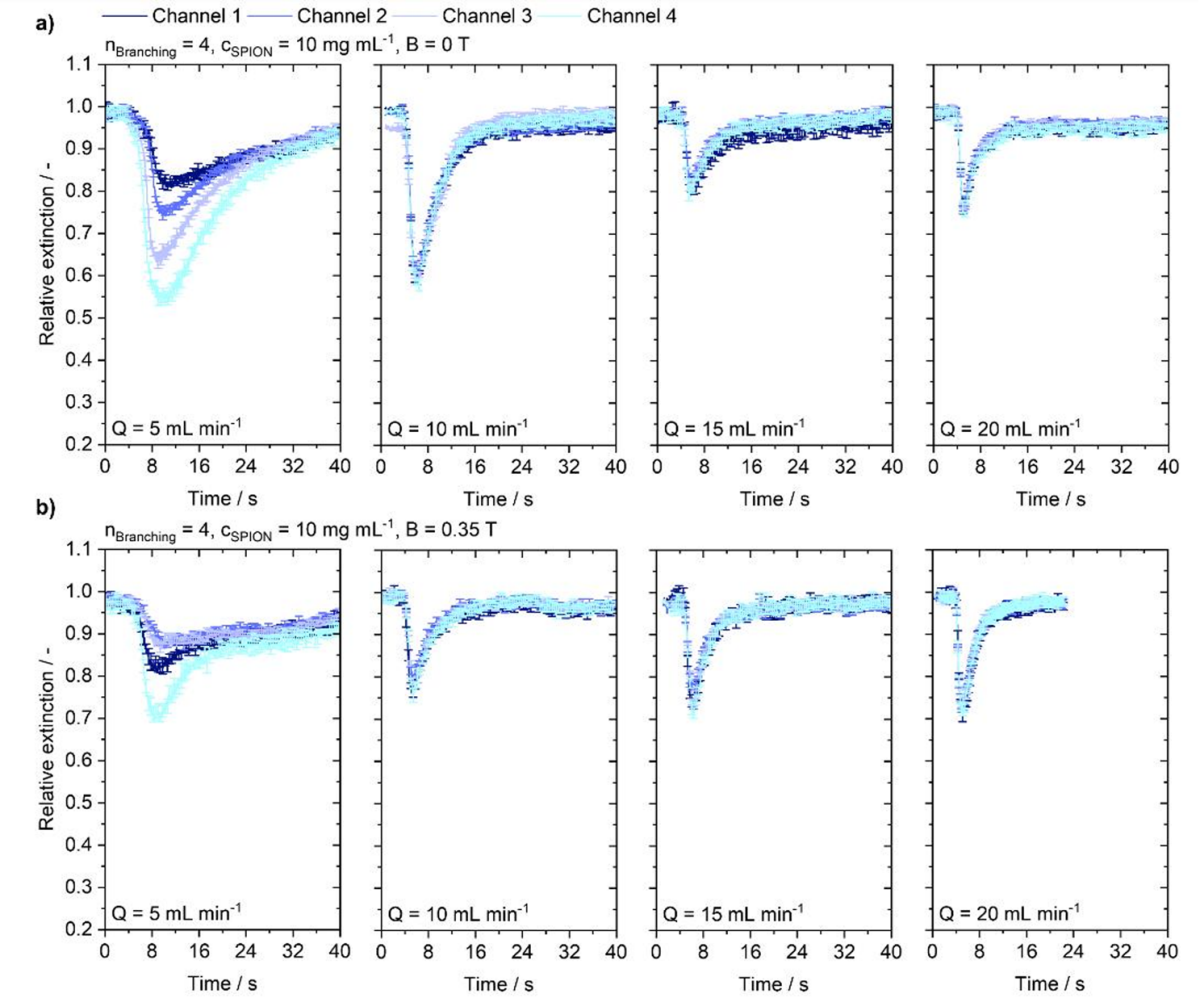
Flow-dependent extinction profile at 4th-order branching injecting a particle suspension with a concentration of 10 mg mL^−1^ into the channel under varying flow conditions of 5, 10, 15 and 20 mL min^−1^. **(a)** Without the influence of a magnetic field. **(b)** Under magnetic steering conditions.

The extinction curves in Figs. 4 and 5, and Fig. 6 qualitatively summarize the temporal propagation behavior for varying branching complexity and flow rates at a constant injected particle concentration of 10 mg mL^− 1^. Distinct peaks occur as particles pass through the straight segments in the center of the specimen. When the flow transports particles through the region of interest, a sharp decrease in relative brightness is observed. After reaching the peak, the particle signal drops and exhibits a tail, indicating slower decay of particles within the channel; this tail is present in all measurements. At high flow rates, the signal declines to the pre-injection level. At a branching order of 2, depicted in Fig. 4, peaks retain this distinct shape across flow rates and magnetic conditions. At 15 and 20 mL min^− 1^, the extinction reaches the same level for all channels. At flow rates of 5 and 10 mL min^− 1^ however, channels 3 and 4 show an increased extinction peak. A slight delay of the peaks in channels 1 and 2 is evident, particularly at 5 mL min^− 1^, indicating delayed particle arrival in the upper channels. With a magnetic field applied, effects are again confined to low flow rates. Also, here the trend of increased particle induced extinction in channels 3 and 4 is observable. However, a higher signal in channel 1 relative to channel 2 suggests that under a magnetic field more particles arrive in the channel closer to the magnet, and the delay of the peak in channel 1 is less pronounced than in channel 2. Also, in the lower channels a relative increase of the peak of channel 4 compared to channel 3 suggests the arrival of more particles in the channel closer to the magnet. The relative increase in extinction in channel 1 and 4 when the magnet is present is explainable by magnetic attraction acting on a sedimented population. Because particles were already biased toward the lower channel regions, the observed difference between channels 1 and 2 should be interpreted as magnetic action on that sedimented fraction rather than capture of particles from across the entire flow cross-section. The recovery of the extinction curve to the pre-injection level suggests that although stratified particle volumes might be affected by a magnetic field, they are not held inside the channels but dragged along by hydrodynamic forces. In higher-order branching structures depicted in Figs. 5 and 6, visible peak broadening in low-flow regimes indicates longer particle persistence in the channels. Without a magnetic field, this is evident in 3rd-order models at 5 mL min^− 1^ (Fig. 5) and in 4th -order models at 5 and 10 mL min^− 1^ (Fig. 6).

At increased flow rates, in both cases no such peak broadening is observed. From Fig. 5, a distinctly broader peak is already evident at 5 mL min^− 1^ and no magnetic field, whereas peaks at elevated flow rates remain visibly narrower. Sedimentation is again visible in the extinction curves and declines with increasing flow rate. Upon application of a magnetic field to the 3rd-order model at 5 mL min^− 1^, the magnet enhances the extinction signal duration according to the previously observed particle sedimentation. The signal is more persistent than without the magnet, and throughout the measurement it does not return to the pre-injection level. Although differences exist between individual channels, the overall behavior is consistent. Channels 2 and 4 in particular show a constant extinction after the brightness drop. In channel 1, a stronger initial drop than without the magnet can be attributed to magnetic attraction into this channel, after which the signal recovers to the same level as channel 2 and remains there for the remainder of the measurement. In channel 3, a slow recovery after the drop is observed, and a persistent accumulation of reduced fractions of SPIONs is evident. Except for channel 1, no distinct peak is observed; instead, the signal remains at a plateau for the remaining time. Again, a shift of the peaks of channel 1 and 4 in comparison to the case without magnet is observable. The visual observation of more particles being present in the two outermost channels is reflected by strong signals in channels 4 and 1 compared to channels 2 and 3. Considering flow rates of 10 mL min^− 1^, a visible magnetic influence remains, which is corroborated by a relative increase of the channel 1-peak with respect to channel 2 and the channel 4-peak compared to channel 3, when comparing conditions with and without magnetic steering, and by the previously observed peak broadening. However, the signal declines normally, indicating that the particles are quickly washed out by the background flow. At 20 and 15 mL min^− 1^, consistent with visual evaluation, no major differences are discernible between conditions without magnetic influence and with magnetic superposition, although a relative increase in the channel 4 signal is still evident. The signal then declines rapidly, showing no enhanced magnetic retention.

The influence of increasing branching complexity on propagation behavior in 4th-order channels is shown in Fig. 6. Distinctly broader peaks are observed at 5 mL min^− 1^. Channels 1 and 2 show a delayed onset relative to the lower channels, consistent with signal delay observed within the other geometries. The delay can also be observed in the images at the moment of particle arrival. While the perturbed cloud at higher flow rates results in a more regular distribution across both daughter channels. Whereas at low flow rates the sedimented particle cloud arrives at the branching confined to the bottom part of the channel and particles propagate on into the lower channel. Only after some time the particles are also entering the upper channel which is seen in a delay of extinction curve onset. The extinction signal of the channels show distinct changes in peak height corresponding to the channel position. With the magnetic field applied, the delay in channel 1 disappears, whereas channel 3 exhibits an enhanced delay. The signal of channel 1 shows a higher extinction than channels 2 and 3, which are on the same level. Extinction in channel 4 however, remains strongest. While the extinction signal does not deteriorate completely, there are no plateaus visible as in the 3rd-order structure. Contrary to the 3rd -order structure, a flow rate of 10 mL min^− 1^, the application of a magnetic field does not lead to visible effects.

**Fig. 7:**
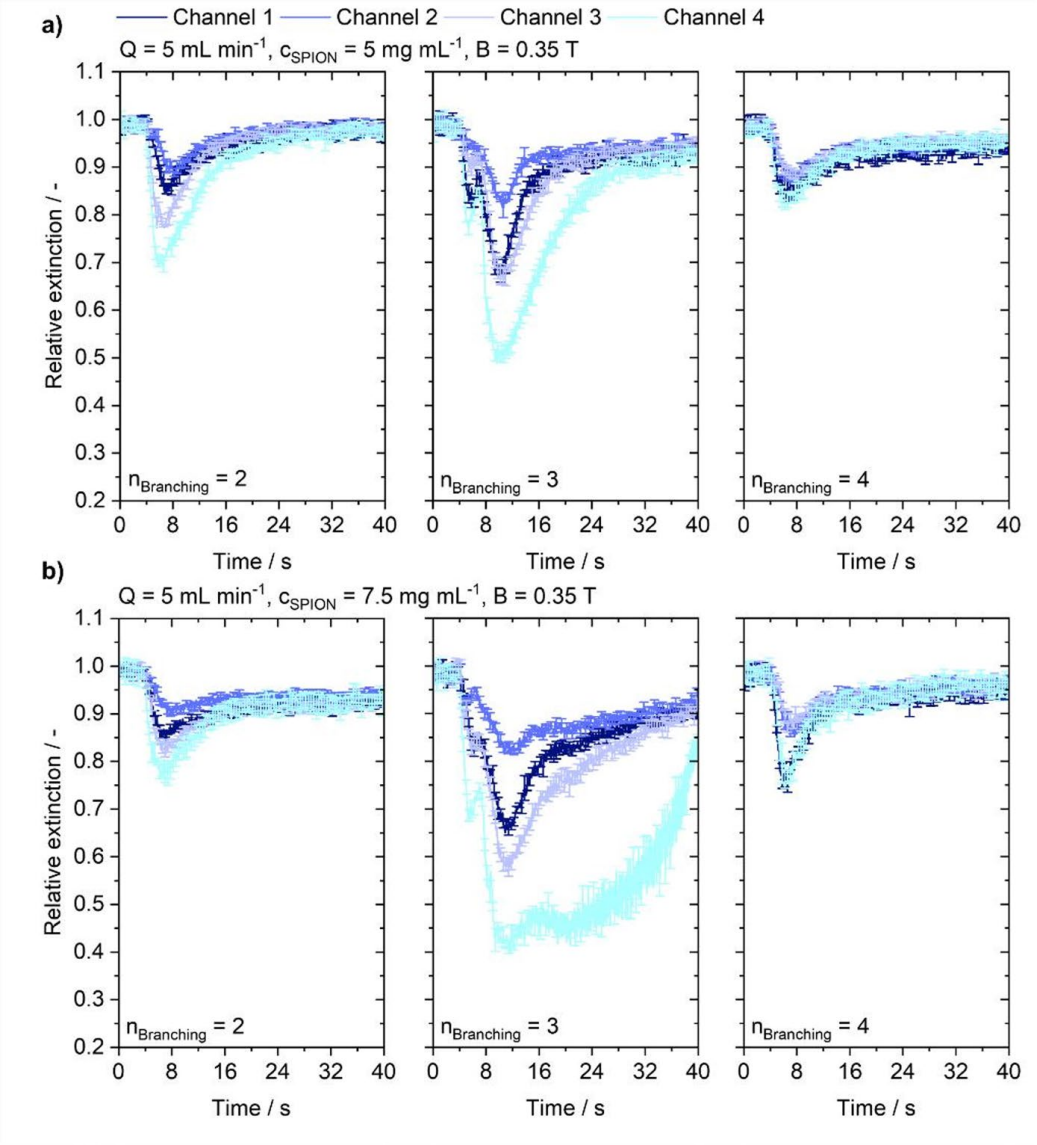
Particle signal for varying branching complexities at injected particle concentrations under the influence of a magnetic field at a background flow rate of 5 mL min^−1^. **(a)** Injected particle concentration of 5 mg mL^−1^. **(b)** Injected particle concentration of 7.5 mg mL^−1^.

Lowering the injected concentration reduces magnetically induced retention overall, although diminished magnetic effects remain where sedimentation concentrates particles. Figure 7 presents extinction curves for injected concentrations of 5 and 7.5 mg mL^− 1^ at a flow rate of 5 mL min^− 1^ under magnetic steering.

Overall, the curves exhibit the same trends as at 10 mg mL^− 1^, although some differences are apparent. With lower injected particle amounts, a sharp decrease in extinction is again observed in all cases, with the strongest signal in channel 4. As discussed previously, this can be attributed to sedimentation of particles in the entering vessel due. The effect is more pronounced in the 2nd- and 3rd-order samples and diminishes in the 4th-order structure. In the 4th -order sample, the peak of channel 3 is relatively small and comparable to that of channel 2, while the channel-1 peak is more pronounced and comparable to channel 4. Considering 3rd-order branching, visible differences emerge. At 7.5 mg mL^− 1^, the observed behavior is found to be similar to that at 10 mg mL^− 1^. However, after reaching a plateau in channel 4, the signal eventually deteriorates, and this deterioration is faster than at 10 mg mL^− 1^. The channel-3 signal also shows a much narrower peak with quicker decay. When the injected concentration is further decreased to 5 mg mL^− 1^, the channel-4 peak exhibits an immediate decline that is slower than in the other channels.

### Injection-induced heterogeneities and concentration gradients

To qualify the boundary conditions at the injection with respect to particle mixture and propagation as a function of the background flow, the feeding tube segment between the injection site and the experimental model was analyzed by video imaging. Images taken 2 s after the injection at background flow rates of 5, 10 and 20 mL min^− 1^ are shown in Supplementary Fig. S3. At a flow rate of 5 mL min^− 1^, a stretched pulsatile velocity profile is visible at the exit of the injection connector. The flow profile is not visibly disturbed, however brightness variations can be observed that can be associated to the pulsatile nature of the flow. Further downstream, the particles segregate and accumulate along the bottom of the channel. At a flow rate of 10 mL min^− 1^, the propagating particles exhibit visible flow disturbances immediately after leaving the injection site. Further downstream, demixing again occurs. At an increased flow rate of 20 mL min^− 1^, the flow does not present a sharp front. Instead, it shows a uniform opaque transverse distribution without visible eddies. Here no significant de-mixing is observed along the flow path. Transport of nanoparticles is shown to be governed by advection-diffusion in several theoretical and experimental studies. In such an advection-diffusion-governed particle transport in laminar flow, it would be expected that no significant sedimentation of isolated nanoparticles appears. Particle behavior would either be governed by advection where particles would follow the stream lines and diffusion would smoothen concentration gradients. Moreso, isolated nanoparticles would experience negligible gravitational settling. However, injection of a highly concentrated nanoparticle suspension can introduce additional mechanisms. At elevated local concentrations, particle aggregation may occur, leading to an increase in effective hydrodynamic size and a reduction in diffusivity. Under such conditions, transport behavior can deviate from that of isolated nanoparticles and exhibit qualitative similarities to sedimentation phenomena reported for micron-scale particles, although the underlying mechanisms differ. The formation of aggregates within the injected volume could therefore represent a plausible explanation for the observed transport behavior. In addition, sharp transient concentration gradients arising during injection of the concentrated nanoparticle suspension may generate Korteweg stresses, leading to additional flow perturbations and enhanced spreading of the particle front. While classical gravitational zone sedimentation is not expected for primary nanoparticles, aggregation and crowding can give rise to zone-sedimentation-like behavior, characterized by collective transport and delayed clearance of particle populations under low-flow conditions.

### Microscopic SPION deposition in additively manufactured models

After five injections of SPIONs during fluidic testing, the particles flowing through the channels showed no visible deposition or accumulation, even when magnetic steering was applied. After sectioning the samples, optical microscopy of the surfaces likewise revealed no particles. In contrast, scanning electron micrographs showed a thin film of small clustered deposits on the surface. These clusters consist of smaller spherical particles with diameters below one hundred nanometers. Deposition occurs on a very small scale, and the clusters are much smaller than the surface pattern generated by the layer wise manufacturing process. Particle deposition appears uniform across the surface and is not influenced by the mesoscale hill and valley topography. Clusters are found both on the hills and in the valleys. In some high magnification micrographs, a thinner and less dense particle layer is visible adjacent to the clusters, although such particle depositions cannot be observed throughout all tested specimens. Its presence indicates a general low-level, non-localized accumulation across the entire surface. Overall, the surface meso- and microstructure do not significantly affect either the amount of accumulation or the spatial distribution of clusters. This holds in particular in the branching region, where the step like surface features from the layer-by-layer process show no detectable influence. High magnification images confirm that clusters form equally across hills and valleys, which supports the conclusion that deposition is independent of process induced surface patterns. Given the small particle size relative to the surface features set by the layer discretization, the surface mesostructure does not play a significant role in SPION deposition. The step at the transition to the bifurcation likewise does not change the accumulation pattern, and neither the clustered morphology nor the particle amount differs from other channel regions.

**Fig. 8:**
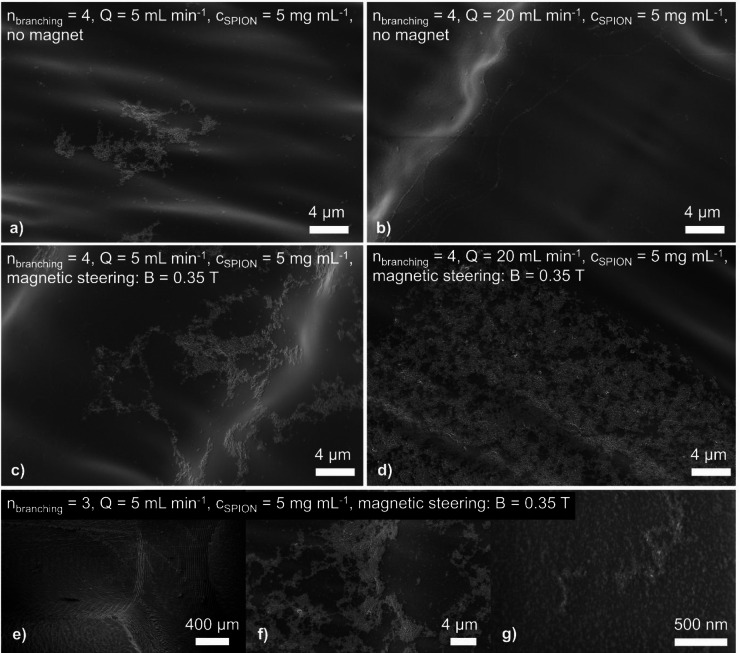
Scanning electron micrographs of SPION depositions observed in proximity to bifurcations. **(a)** 4th-order bifurcation after injection of 5 mg mL^− 1^ SPIONs with 5 mL min^− 1^ background flow and no magnetic field applied. **(b)** 4th-order bifurcation after injection of 5 mg mL^− 1^ SPIONs with 20 mL min^− 1^ background flow and no magnetic field applied. **(c)** 4th-order bifurcation after injection of 5 mg mL^− 1^ SPIONs with 5 mL min^− 1^ background flow under the influence of a magnetic field. **(d)** 4th-order bifurcation after injection of 5 mg mL^− 1^ SPIONs with 20 mL min^− 1^ background flow under the influence of a magnetic field. **(e-f)** 3rd-order bifurcation after injection of 5 mg mL^− 1^ SPIONs with 5 mL min^− 1^ background flow under the influence of a magnetic field.

## Discussion

### Macroscopic transient nanoparticle distribution

Comparison of particle distributions across the investigated flow rates indicates that gravity-driven sedimentation increasingly influences particle propagation at low flow velocities. The sedimentation observed at 5 mL min^− 1^ implies that gravity substantially drives the particles toward the lower channel region even over distances of a few cm. This stratification becomes less pronounced as flow rate increases, suggesting that injection-induced disturbances through pulsatile flow and enhanced convective mixing reduce the persistence of sedimentation-driven concentration gradients. At 20 mL min⁻¹, particle distributions appear largely homogeneous across channels for all tested conditions. This could be explained by stronger mixing during and after injection, which can suppress sedimentation-driven stratification. Differences between low and high flow regimes show that flow conditions strongly influence particle propagation and can affect the sedimentation effects. This is corroborated by the development of pixel extinction at specific points within the channel. The return of the signal to the pre-injection level at high flow rates indicates that no particles remain trapped within the channel and that wall accumulation does not cause a persistent post-injection increase in extinction. All channels showing the same level of extinction indicates a homogenous particle distribution in the network and suggests that flow conditions dominate particle distribution at high flow rates. At 5 and 10 mL min⁻¹, all geometries exhibit pronounced stratification, reflected by higher extinction signals in channels 3 and 4. The observed delay of the peaks in channels 1 and 2, particularly at 5 mL min^− 1^, indicates delayed particle arrival in the upper channels. This delay could be explained by particle sedimentation and the resulting creeping flow profile. Under low flow rates, particle distribution is non-uniform due to particle settling toward the lower region of the channels. The observed relative increase in extinction in channel 1 and 4 during magnet exposure at 5 and 10 mL min^− 1^ likely reflects the magnet’s action on this pre-existing, locally enriched particle layer. This observation indicates that the magnetic gradient is able to modify the distribution of an already stratified particle cloud. At the measured conditions, the magnetic field seems to locally modify a settled particle distribution but does not fully counteract fluidic forces across the channel. At high flow rates, perturbances at the injection lead to good mixture and minimize both sedimentation and magnetic effects. While the flow rate clearly affects particle distribution in 2nd-order structures, it does not substantially alter the duration of the particle signal. The peak broadening observed in higher-order branching structures without the influence of a magnetic field indicates longer particle persistence in the channels. However, this effect is only observed for low flow rates, which indicates that higher branching complexity does not diminish the dominance of hydrodynamic forces under high-flow conditions. A longer duration of the SPION signal reflects the influence of branching complexity through changes in flow velocities and fluidic resistance. As these effects diminish with increasing flow rates, rising flow velocity appears to be the dominant driver of particle transport. In the 3rd-order structures at 5 mL min^− 1^ particle sedimentation is again visible, while the broad peaks indicate a high particle retention in the channel network. Upon application of a magnetic field at 5 mL min^− 1^ the particles appear to be attracted into the outer channels 1 and 4. This is also evident by a visible shift in the onsets of the extinction curves in the respective channels. The distinct persistence of the signal in channel 4 throughout the measurement indicates an extended particle residence time under magnetic field influence. However, it is evident that the particle persistence in channels 3 and 4 benefits from the sedimentation-induced increase in local particle concentration, while the major amount of particles attracted into channel 1 is washed out again. These data indicate that sedimentation continues to bias the particle distribution toward specific channels. Application of the magnet produced a transient increase in particle flux into channel 1, but the majority of these magnet-redirected particles were removed by the background flow during the course of the experiment. This behavior implies that the magnetic force acted on the sedimentation-induced particle cloud. Considering flow rates of 10 mL min^− 1^, a visible magnetic influence remains, suggested by the development of the extinction peaks as well as peak broadening corresponding to longer particle retention. However, no particle trapping is observed, and the signal declines normally. While the 4th-order structures also show an enhanced particle persistence at 5 mL min^− 1^, it is not as pronounced as that observed for the 3rd-order structures. Unlike the 2nd- and 3rd-order structures, particle sedimentation in the 4th-order model are evident only at 5 mL min^− 1^, with differences in peak extinction reflecting channel positions. Visible shifts in the delay of the channel signals are again demonstrating the superposition of the temporal, sedimentation-driven propagation with magnetic influence. Extinction in channel 4 remains strongest, which indicates a persistent gravitational contribution to the observed time-extinction profiles. A more pronounced channel 1 peak appears when the magnet is present. At low flow rates this peak may represent magnetically perturbed particles; however, at higher flow rates the peak is not indicative of substantial magnetically driven re-distribution. Considering a flow rate of 10 mL min^− 1^, no sedimentation effects are observable and also the application of a magnetic field does not yield changes. Magnetic forces thus alter, but do not dominantly govern, particle propagation under the applied flow conditions and the resulting particle stratification. Moreso, it is implied that locally enriched particle populations appear to be a prerequisite for observable magnetic effects to occur under the present conditions. This is also suggested by the evaluation of reduced injected particle concentration. When the particle concentration is reduced, under magnetic steering the curves exhibit the same trends as at 10 mg mL^− 1^, although some differences are apparent. Particles still show primarily sedimentation. However, the effect diminishes in the 4th-order structure. The relative sizes of the signal in channels 3 and 2 indicate a magnetic effect on the sedimented particles in the 3rd- and 4th-order samples, consistent with the observations at 10 mg mL^− 1^. In the 4th-order branching, a remaining tail indicates persistence of SPIONs after injection. Reducing particle concentration reduces the magnitude and duration of particle extinction in some geometries. However, where sedimentation amplifies particle concentrations locally, residual magnetic influences may still remain observable. In the 3rd-order structures, lower injected concentrations lead to a more rapid decay of the extinction signal compared to the case of 10 mg mL^− 1^, indicating reduced particle persistence within the network. These observations suggest that both the injected particle amount and the topology-dependent, sedimentation-induced particle distribution influence the macroscopic persistence of particles during magnetic steering. Importantly, these effects appear only under conditions where particle stratification is already present, and they cannot be attributed to magnetic forces acting on isolated nanoparticles.

Accordingly, the present results support the qualitative magnetic effect on sedimentation-stratified particle populations in branched channel networks, while the steering of well-mixed or strongly disturbed flows at elevated velocities remains ineffective.

The present observations are consistent with previous studies on magnetic drug targeting, which indicate that effective magnetic manipulation under flow conditions typically relies on collective particle effects rather than on isolated nanoparticles. Recent work by Mosavi et al. demonstrated that enhanced magnetic targeting efficiency is achieved through formulation strategies and particle populations exhibiting increased effective magnetic susceptibility. Complementary theoretical analyses by Sharma et al. have shown that magnetophoretic forces acting on individual nanoparticles are generally insufficient to overcome hydrodynamic drag under physiologically relevant flow conditions. Moreover, studies on particulate systems, such as those reported by Ardalan et al., highlight how aggregation and clustering can substantially amplify magnetic responsiveness. In this context, the present results provide experimental evidence that sedimentation-induced stratification and local particle enrichment represent key prerequisites for observable magnetic effects in branched flow networks under moderate field gradients^7–9^.

To directly assess whether magnetic forces can compete with hydrodynamic drag under the present experimental conditions, a force-balance estimate was performed. For a singular isolated particle of volume $\:{V}_{P}$ and susceptibility $\:\chi\:$, that does not experience magnetic dipole – dipole – interactions, particle – particle interaction forces or particle – wall – interaction, the magnetophoretic force acting on a particle in a magnetic field is given by Eq. 5^47–49^.

5 $$\:\left|{\boldsymbol{F}}_{mag}\right|=\:\frac{{V}_{P}\:\chi\:}{{\mu\:}_{0}}\:\left|\boldsymbol{B}\right|\:\nabla\:\left|\boldsymbol{B}\right|$$

Based on this equation a rough approximation was made on the force a single particle would experience by the applied magnetic field in the outermost branch closest to the magnet. A particle with a radius of 40.45 nm and a susceptibility of 0.0061794 would experience a force of $\:1.14\times\:{10}^{-18}$ N, $\:7.93\times\:{10}^{-19}$ N and $\:8.99\times\:{10}^{-19}$ N in the 2nd -, 3rd - and 4th -order samples, respectively. These values were compared to the hydrodynamic forces acting on the particle. For small spheres of radius $\:r$ in a laminar flow of a liquid of viscosity $\:\eta\:$, and velocity $\:{u}_{f}$ viscous forces dominate and the drag force $\:{F}_{drag}$ acting on the particle of velocity $\:{u}_{p}$ is given by Eq. 6^51,52^:

6 $$\:{F}_{mag\:}+{F}_{drag}=0,\:\:\left|{\boldsymbol{F}}_{drag}\right|=\:6\pi\:\eta\:r({u}_{p}-{u}_{f})$$

Accordingly, the magnetophoretic force induces a terminal slip velocity between particle and fluid:

7 $$\:{u}_{mag}=\:\frac{{F}_{mag}}{6\pi\:\eta\:r}$$

Using the estimated magnetic forces and the viscosity of the background fluid, this magnetophoretic velocity $\:{u}_{mag}$ was calculated. In all cases, the resulting velocity is in the order of 1 nm s^− 1^ and several orders of magnitude smaller than the characteristic flow velocities in the outer branches retrieved from the continuity equations which are in the order of tens of mm s^− 1^. This comparison demonstrates that, under the applied magnetic field gradients and flow conditions, isolated SPIONs are expected to follow the streamlines of the flow with negligible magnetic cross-stream migration. Similar force-balance considerations have been reported previously, demonstrating that magnetophoretic forces on isolated nanoparticles are typically several orders of magnitude smaller than viscous drag under laminar flow conditions, thereby limiting single-particle steering to extremely high magnetic field gradients^8^. Consequently, given the applied hydrodynamic SPION diameters, the steering of singular, non-interacting SPIONs is physically infeasible within this setup. However, steering effects of macroscopic non-disturbed, stratified flows, emerges as a feasible possibility. As clusters aggregate, the magnetic force scales with the volume ($\:{F}_{mag}\propto\:\:{r}^{3}$) of the aggregate, while drag force scales with just the radius ($\:{F}_{drag}\propto\:\:r$). So, particle clustering in stratified volumes would be beneficial to magnetic steering. Considering particle interaction forces, weak colloidal stabilization, van der Waals attraction, or compression of electrostatic double layers can promote the formation of small aggregates even in the absence of a magnetic field^53^. In magnetic fields, induced dipole–dipole interactions may further contribute to the alignment of such pre-existing clusters, although this mechanism was not directly resolved in the present experiments^54^. These mechanisms provide possible explanations for the observed macroscopic steering of sedimentation-stratified particle volumes despite the negligible magnetophoretic force acting on individual SPIONs. This, in turn, indicates the preferable avoidance of the disturbance of injected SPION flows in order to prevent dispersion and maintain the steerability. Successful steering of single particles would only be achievable with very high magnetic fields. However, an enhanced understanding of the formation and motion of aggregates and stratified particle volumes in branched channel structures may inform future strategies aimed at exploiting collective particle effects under controlled conditions.

In the discussed experimental work, for a proof-of-concept distilled water was used as background fluid to serve as a simplified Newtonian reference. Here geometric, magnetic and inertial effects are separated from biological influences. This has implications for the comparison of the experimental results obtained in the present study with particles injected into real blood. The presence of cells and proteins in real blood as well as the shear thinning behavior of blood exhibits influences on the particle propagation by cell-mediated dispersion and particle-particle-interactions, affecting particle cluster formation^52^. However, while relevant in small arteries and the vascular bed, in large proximal arteries the size of several hundred µm, fluidic effects such as shear thinning or the Fåhræus–Lindqvist effect can be neglected in bigger arteries encountered in the proximal tumor feeding system^55,56^. On the other hand, increased viscosity of blood compared with distilled water have implications on the particle propagation behaviour through increased drag affecting particle mobility. Especially the particle sedimentation observed in this study would be less pronounced in a liquid of higher viscosity^26^. Though it was observed that sedimentation-stratified particle volumes show response to low field strength, low gradient magnetic fields, care has to be taken when trying to extrapolate the observations to biological systems. As sedimentation of particles can be expected to be lower, the observed effects of gravity-induced particle stratification enhancing magnetic responsiveness are not directly transferable^57^. However, it could be shown that particle-particle-interactions resulting in cluster formation through local particle stratification, are a significant effect that can be exploited for more effective steering strategies, though in blood rather than gravity-induced effects, cell-mediated effects and magnetic clustering might be more pronounced^53,57^. Further experimental work should consider the higher viscosity of blood and the presence of cellular contents to provide a more thorough picture of particle propagation depending on vascular topology and injection conditions.

### Injection-induced heterogeneities and concentration gradients

Comparing the particle flow two seconds after injection reveals a clear dependence of particle propagation on the background flow rate. At 5 mL min^− 1^ the formation of a pulsating flow profile indicates laminar conditions in the tube downstream of the injection. The higher velocity of the injected particle stream does not appear to generate significant disturbance when the jet impinges on the opposing wall in the T connector. Brightness variations within the particle stream arise from the peristaltic pump pulsation. Further downstream, the particles segregate and arrive accumulated along the bottom of the channel at the first bifurcation. This behavior points to rapid segregation of the SPION suspension driven by the density difference between the distilled water of the background flow and the injected SPION suspension. Such density differences are described as possible cause for particle segregation under laminar flow^58^. Even in absence of inertial instabilities, a sharp density difference with the background fluid can cause sedimentation along the flow path through development of a relative slip velocity. This separation could explain the concentration differences observed between different channels. The induction of disturbances at higher background flow rates, which is observed in Supplementary Fig. S3, promotes mixing of the particle suspension with the background fluid. Overall, the onset and development of disturbance in the mixed flow are governed by the background flow. A plausible mechanism is the increase in pulsation amplitude produced by the peristaltic pump. Higher pulsations and pressure fluctuations, together with the disturbance caused by the injected jet, favor the induction of unsteady flow conditions, whereas lower flow rates facilitate re-stabilization toward laminar conditions^59,60^. The background flow rate furthermore correlates with particle spreading immediately after injection. At 5 mL min^− 1^ the particles spread most broadly, so the entire particle cloud requires more time to pass the observation point. With increasing flow rate, the extinction peak becomes progressively narrower. This highlights the role of local flow velocity and the associated inertial forces in particle transport, with a positive correlation between low flow velocity and extinction peak width. This aspect is essential for magnetic particle steering, since particles travel more slowly in low flow regimes, which increases the time during which the magnetic field can act on them. Increased particle volume fractions near the bottom of the channel further amplify the magnetic force on the particle cloud, enhancing the effectiveness of magnetic control over this near wall aggregation. Consistent with these mechanisms, mixing at high background flow leads to a more homogeneous particle distribution in the channel structures. The comparatively low level of sedimentation along the flow path under these conditions is supported by the increased particle speed, which reduces the fraction of particles that segregate. However, it should be mentioned that the pulsation frequency of the pump is out of bounds of any physiological pulsation that the tumor feeding system would experience. Consequently, the observed injection-induced disturbances and emerging mixing effects represent an experimental artifact of pulsatile flow perturbations that would not be observed in a physiological system, as actual in vivo conditions are expected to exhibit weaker pulsation-driven mixing and a stronger dominance of steady laminar transport.

#### Microscopic SPION deposition in additively manufactured models

These observations on particle surface accumulation obtained by the SEM suggest that, under the applied flow conditions, macroscopic surface patterns do not induce significant local flow disturbances and laminar flow remains predominant. Other factors, such as locally reduced flow velocities, are therefore more likely to govern the observed behavior. Figure 8 shows representative surface images under different experimental conditions. At a low flow rate of 5 mL min^− 1^, non-localized clustered accumulation is present on the surface. Magnetic steering, considering the applied conditions of a locally homogeneous field of B = 0.35 T, does not appear to alter the extent or pattern of accumulation. When the flow rate is increased to 20 mL min^− 1^ without magnetic steering, visibly fewer SPIONs are deposited on the channel surface. This behavior can be explained by the higher wall shear stress at elevated flow rates and aligns with reports of reduced particle accumulation on walls under stronger shear. In addition, creeping flow caused by the observed enhancement of particle concentration in the lower parts of the samples, may further enhance particle interactions with the wall. Under magnetic steering in Fig. 8b, the formation of non-localized clusters becomes more pronounced again. This could explain enhanced non-localized particle deposition. However, in neither of these cases any localized particle accumulation caused by the magnet is observed.

### Conclusion and outlook

In the present work, experiments on the transport of super paramagnetic iron oxide nanoparticles were performed based on generalized, statistically biomimetic blood vessel-inspired geometries. Relying on empirical studies on the branching behavior as well as based on empirical models, a generalized vascular-inspired 3D-printed model was derived that allows for the cross-individual, generalized investigation of nanoparticle deposition and steering processes. Using a newly developed fluidic test bench, the flow-dependent propagation behavior of the particles was observed by means of optical measurements. Localized particle adhesion was further analyzed by SEM.

Force estimates show that under the magnetic field gradients applied in this study, the magnetophoretic force acting on an isolated SPION is several orders of magnitude smaller than viscous resistance, resulting in a negligible magnetically induced drift indicating that steering of isolated particles is not feasible within the present experimental configuration. Nevertheless, the experimental findings show macroscopic differences in particle extinction and residence behavior were observed under magnetic actuation at low flow rates. These effects occur only when particle populations are already stratified by sedimentation and are suppressed in disturbed or well-mixed flow regimes. The observations indicate that magnetic fields primarily influence the transport of locally enriched particle populations rather than individual nanoparticles. Aggregation or cluster formation may contribute to the enhanced magnetic responsiveness of such populations, although these mechanisms were not directly resolved in this work. Overall, the results demonstrate that particle concentration, network topology, and flow regime critically govern the emergence of macroscopic magnetic effects in branched channel systems. These findings align with recent magnetic drug targeting studies emphasizing the necessity of collective particle effects, aggregation, or carrier-based strategies to achieve meaningful magnetic manipulation under flow conditions, rather than direct steering of isolated nanoparticles. SEM imaging of the surface after flow experiments reveals a macroscopically homogeneous particle distribution and low, non-localized clustering on the channel walls. The deposition pattern of thin particle layers alongside randomly distributed particle clustering is found to form well below the size of additive manufacturing induced surface patterns, originating in the discretized layer-by-layer manufacturing. This suggests a subordinate dependence of process specific structures on SPION deposition, while the influence of flow-induced disturbances is found to be of considerable significance to the efficacy of magnetic steering.

## Supplementary Information

Below is the link to the electronic supplementary material.

Supplementary Material 1

## Data Availability

The data evaluated in this work are available upon reasonable request from the corresponding author.
