# Supplementary material for: Generalized blood vessel models for magnetic nanoparticle-based oncology: geometric and microfluidic properties
Source: Sci Rep. 2026 Jan 27;16:3701. doi: 10.1038/s41598-026-37348-7 (PMC12852665; doi:10.1038/s41598-026-37348-7)
Supplement: Supplementary file 1 — Supplementary Material 1 [file 41598_2026_37348_MOESM1_ESM.pdf]

Supplementary Information on “Generalized blood vessel models for magnetic nanoparticle-based oncology: geometric and microfluidic properties”

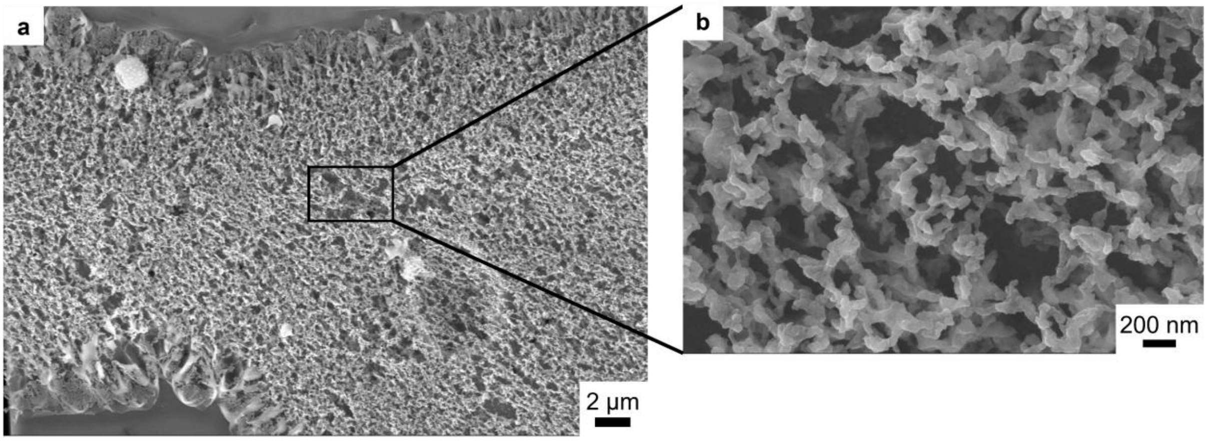

Supplementary Figure S1: *Scanning electron micrographs of the SPION clustering on an object slide upon drying.*

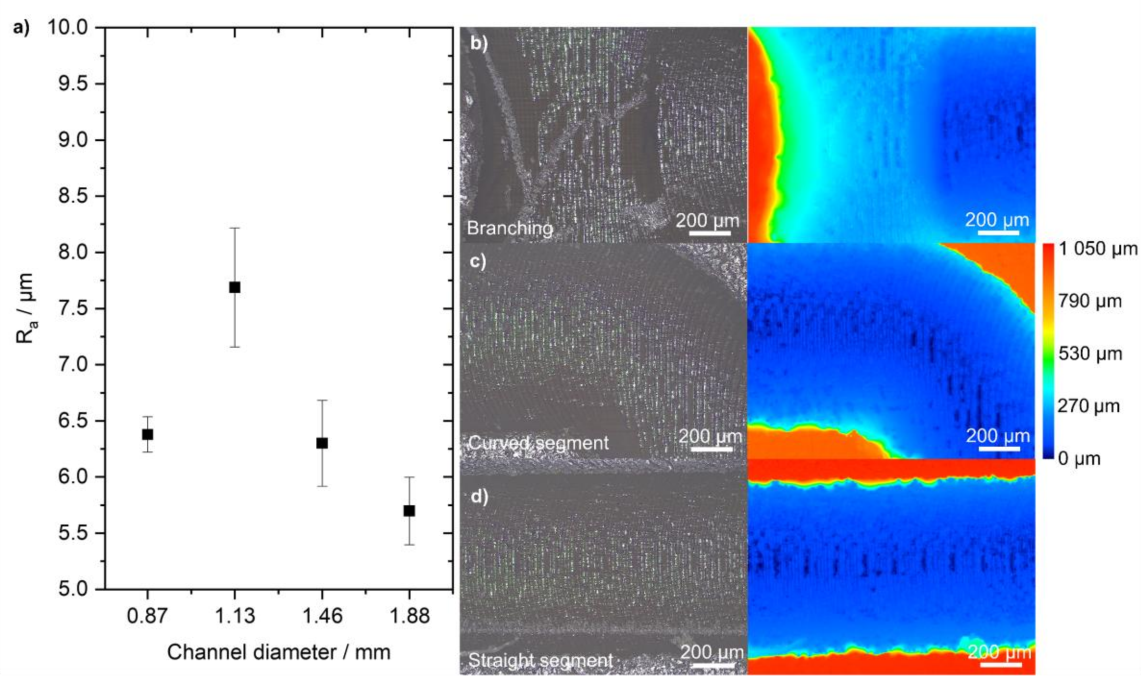

Supplementary Figure S2: *Evaluation of the surface quality in additively manufactured channel structures.* ***a)*** *Surface roughness evaluation of straight segments for different channel sizes.* ***b-d)*** *Expemplary images (laser + optical, left) and derived height images (right) of the surface structure of the 3rd order bifurcation, a curved segment (**c**) and a straight segment (**d**) in the channel following the bifurcation (**b**).*

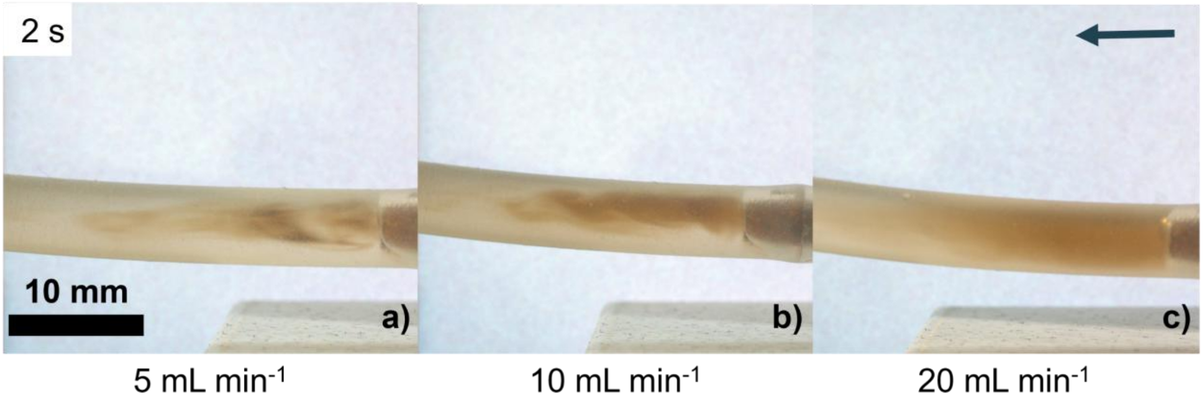

Supplementary Figure S3: *Flow-dependent nanoparticle transport 2 s after the injection at varying background flow rates. The arrow indicates the direction of flow.* ***a)*** *5 mL min^{-1}* ***b)*** *10 mL min^{-1}* ***c)*** *20 mL min^{-1}.*

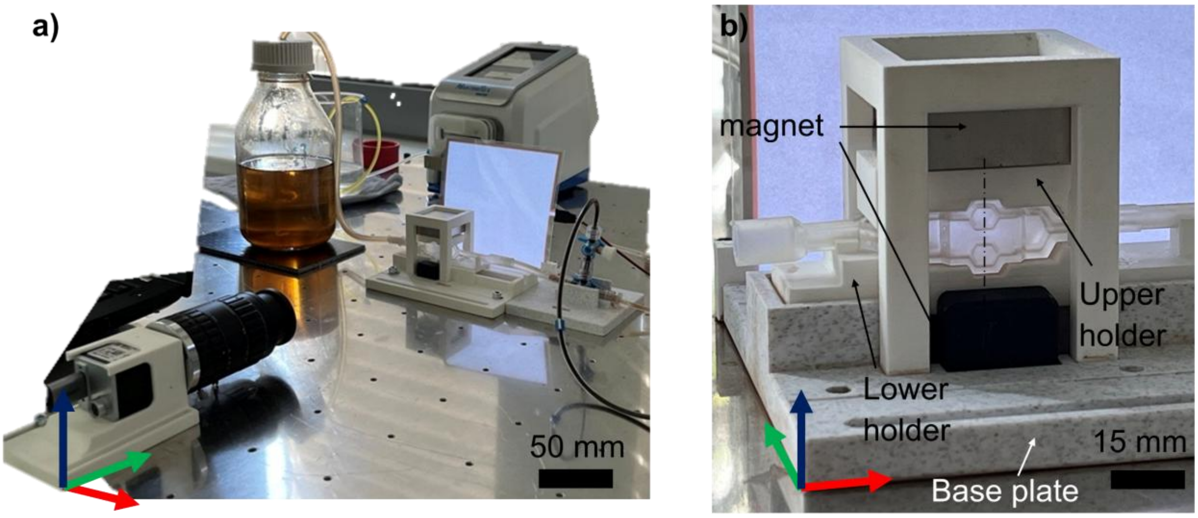

Supplementary Figure S4: *Images of the test bench setup and specimen fixture.* ***a)*** *Overview of the testbench setup reflecting the relative position of the camera system and the samples.* ***b)*** *Image of the sample fixture specifying the base plate and sample holders. The position of the sample relative to the magnets is provided by interlocking the baseplate and sample holder.*

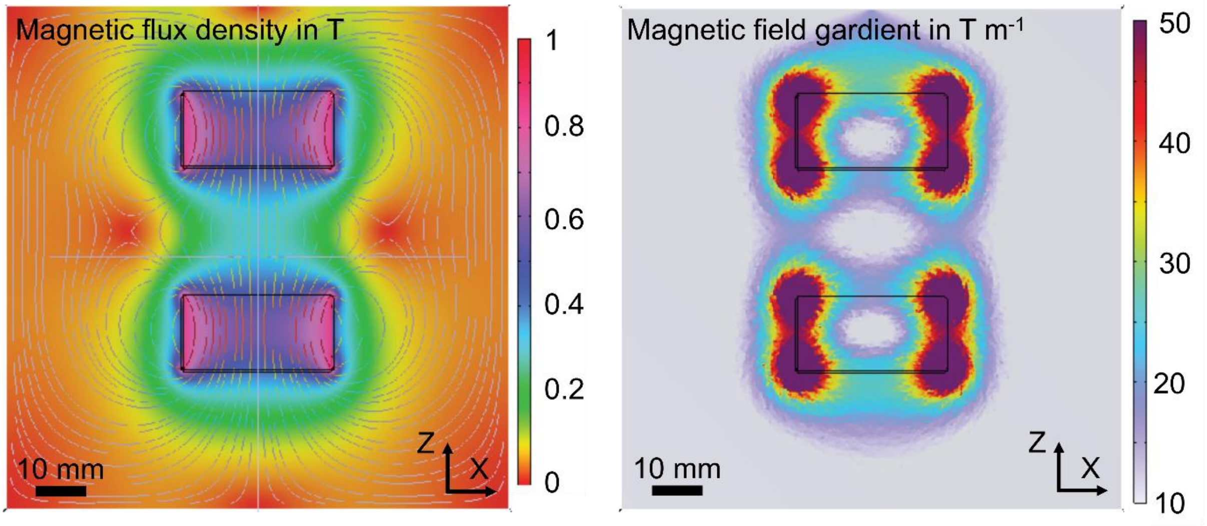

Supplementary Figure S5: *Field plots of the magnetic flux density B and the grad B of the magnetic setup used in the present work.*

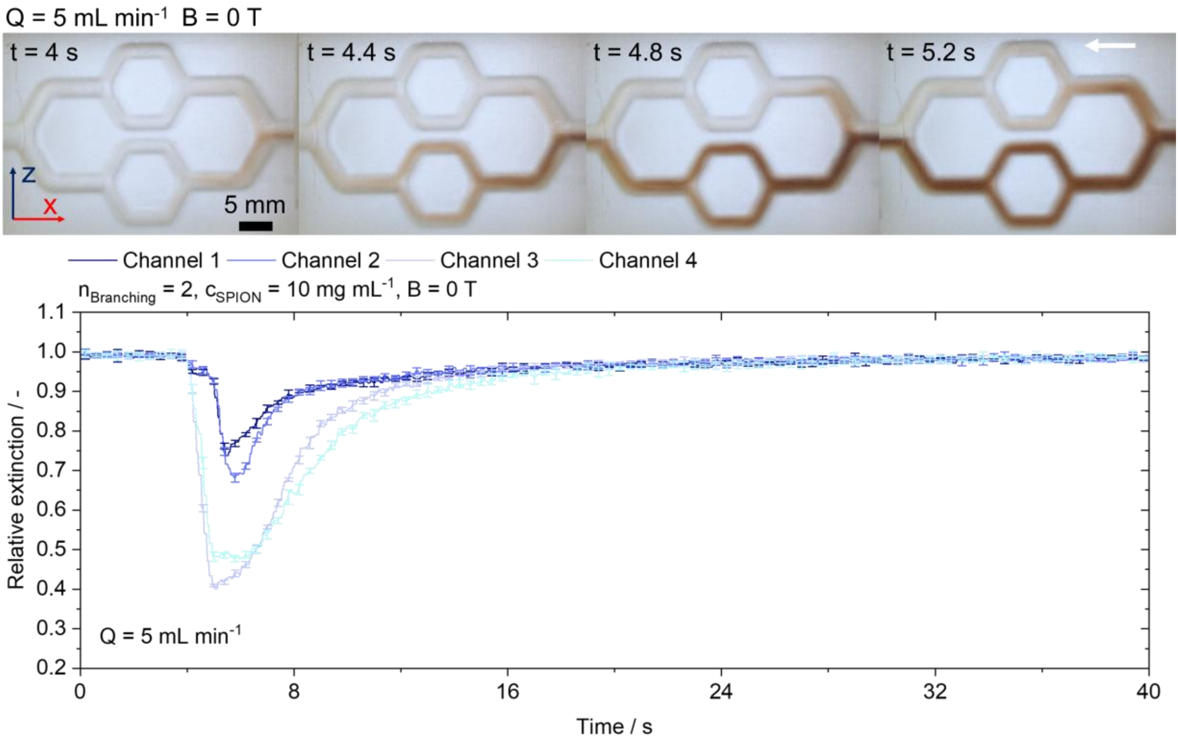

Supplementary Figure S6: Particle distribution and propagation in the 2nd-order channel structures at the flow rate of 5 mL min^{-1} without the influence of a magnetic field. *The images show the particle distribution at different time steps, showing the stratification-induced preference for the lower channels. The corresponding relative extinction curves visualize the location dependent particle arrival.*

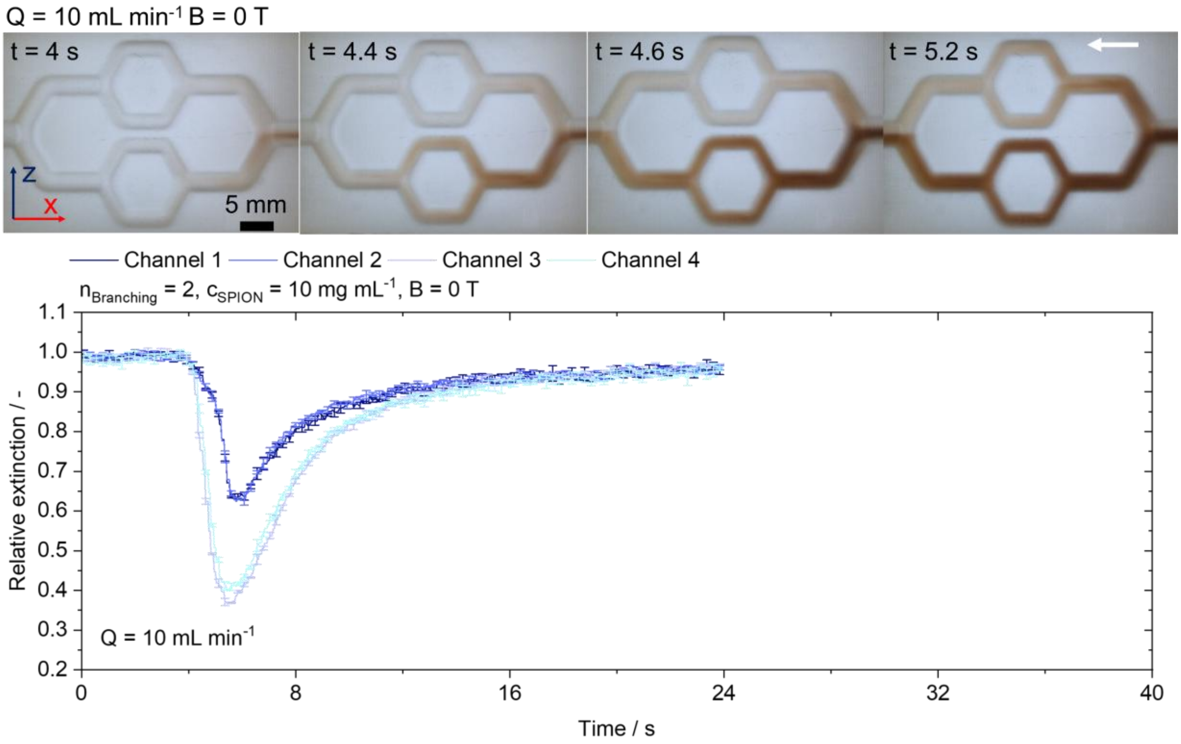

Supplementary Figure S7: Particle distribution and propagation in the 2nd-order channel structures at the flow rate of 10 mL min^{-1} without the influence of a magnetic field*. The images show the particle distribution at different time steps, showing the stratification-induced preference for the lower channels. The corresponding relative extinction curves visualize the location dependent particle arrival.*

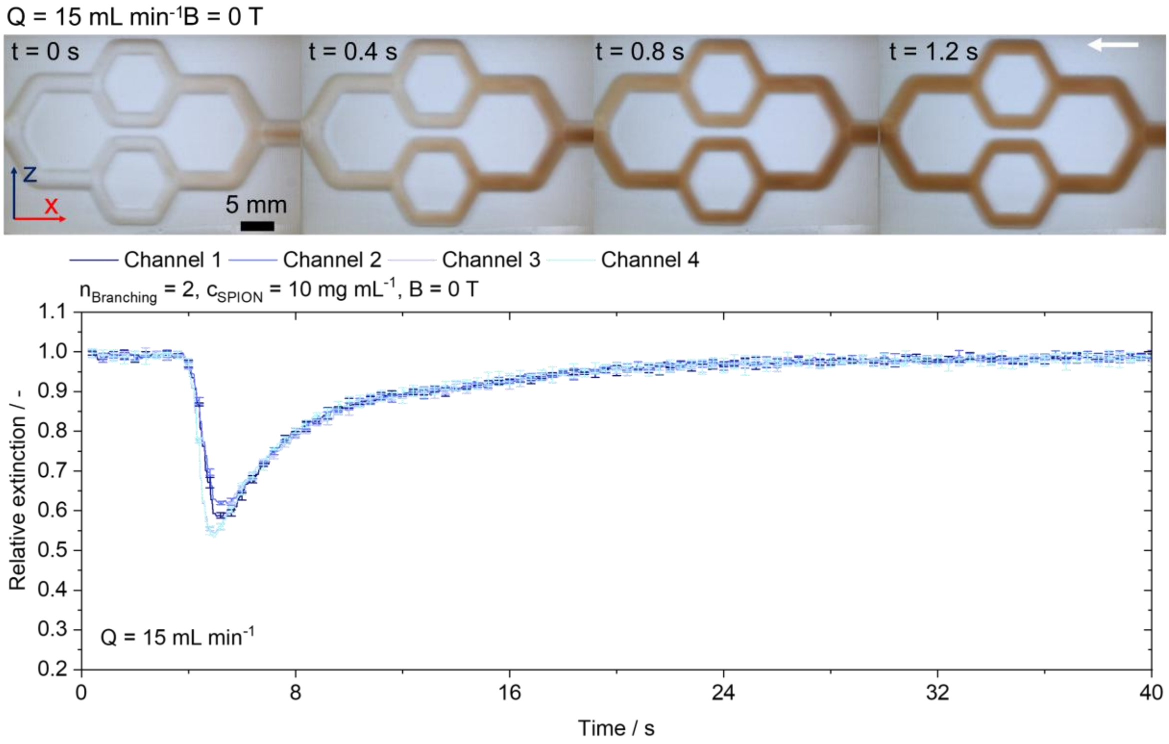

Supplementary Figure S8: *Particle distribution and propagation in the 2nd-order channel structures at the flow rate of 15 mL min^{-1} without the influence of a magnetic field. The images show the particle distribution at different time steps with mixing and a therefore more homogenous particle distribution becoming more prominent. The corresponding relative extinction curves visualize the location dependent particle arrival.*

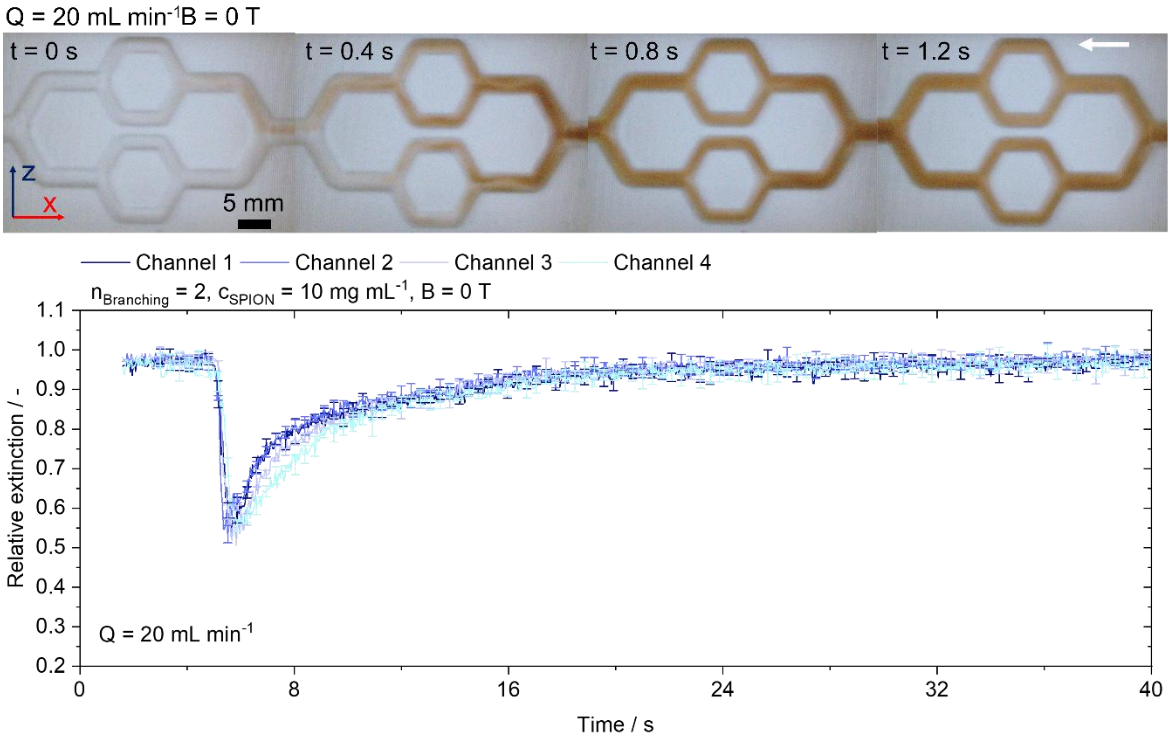

Supplementary Figure S9: *Particle distribution and propagation in the 2nd-order channel structures at the flow rate of 20 mL min^{-1} without the influence of a magnetic field. The images show the particle distribution at different time steps with mixing and a homogenous particle distribution being evident. The corresponding relative extinction curves visualize the location dependent particle arrival.*

**Branching generation**	**0**	**1**	**2**	**3**	**4**
Segment radius	1,22 mm	0,942 mm	0.729 mm	0.563 mm	0.436 mm
Segment length	12 mm	9.3 mm	7.21 mm	5.59 mm	4.33

Supplementary Table S1: *Channel dimensions of the models calculated from the relationships described in equations 2 and 4, assuming a radius exponent α = 2.7 and an exponent κ = 1.*

**Flow rate**	**5 mL min^{-1}**	**10 mL min^{-1}**	**15 mL min^{-1}**	**20 mL min^{-1}**
Pump RPM	32.97	65.93	98.9	131.87
Stroke frequency	197.82 min^{-1}	395,58 min^{-1}	593.4 min^{-1}	791.22 min^{-1}

Supplementary Table S2: *Pump RPMs and resulting stroke frequency of the peristaltic pump for each of the applied flow rates.*

	distance	|B|	grad(|B|)	F_mag		v_fluid	v_mag	
2 gen	5.08 mm	0.371	18	1.14E-18	N	0.0499	1.49E-09	m s^{-1}
3 gen	5.96 mm	0.358	13	7.93E-19	N	0.042	1.04E-09	m s^{-1}
4 gen	7.52 mm	0.337	15.66	8.99E-19	N	0.03484	1.18E-09	m s^{-1}
Particle properties								
diameter	40.45 nm	4.05E-08	m					
volume		3.47E-23	m^3					
susceptibillity		0.0061794						
Constants								
μ_0		0.000001257						
viscosity		1.00E-03	Pa s					

Supplementary Table S2: *Calculation of magnetophoretic force and magnetophoretic velocity.*
